# Semiconductor quantum computation

**DOI:** 10.1093/nsr/nwy153

**Published:** 2018-12-22

**Authors:** Xin Zhang, Hai-Ou Li, Gang Cao, Ming Xiao, Guang-Can Guo, Guo-Ping Guo

**Affiliations:** 1 Key Laboratory of Quantum Information, CAS, University of Science and Technology of China, Hefei 230026, China; 2 Synergetic Innovation Center of Quantum Information & Quantum Physics, University of Science and Technology of China, Hefei 230026, China

**Keywords:** semiconductor quantum dot, qubit, quantum computation, spin manipulation

## Abstract

Semiconductors, a significant type of material in the information era, are becoming more and more powerful in the field of quantum information. In recent decades, semiconductor quantum computation was investigated thoroughly across the world and developed with a dramatically fast speed. The research varied from initialization, control and readout of qubits, to the architecture of fault-tolerant quantum computing. Here, we first introduce the basic ideas for quantum computing, and then discuss the developments of single- and two-qubit gate control in semiconductors. Up to now, the qubit initialization, control and readout can be realized with relatively high fidelity and a programmable two-qubit quantum processor has even been demonstrated. However, to further improve the qubit quality and scale it up, there are still some challenges to resolve such as the improvement of the readout method, material development and scalable designs. We discuss these issues and introduce the forefronts of progress. Finally, considering the positive trend of the research on semiconductor quantum devices and recent theoretical work on the applications of quantum computation, we anticipate that semiconductor quantum computation may develop fast and will have a huge impact on our lives in the near future.

## INTRODUCTION

Recently, the tremendous advances in quantum computation have attracted global attention, putting this subject again in the spotlight since it was first proposed by Richard Feynman [1] in 1982. In the race to build a quantum computer, several competitors have emerged, such as superconducting circuits [2,3], trapped ions [4,5], semiconductors [6,7], nitrogen-vacancy centers [8,9], nuclear magnetic resonance [10], etc. Among these, semiconductors are a powerful contender for their significant role in the field of classical computing. They have not only changed our lives with the personal computer, smartphone, Internet and artificial intelligence but also boosted economics worldwide, such as the birth of Silicon Valley in the USA. With the aim of promoting another technological revolution in the quantum field, in the last decade, several significant breakthroughs in quantum information processing have been made based on semiconductors. These advances in turn confirm the faith of researchers trying to build a quantum computer out of semiconductors.

Similar to the classical counterpart that is built upon classical bits, a quantum computer is made of quantum bits, which are also called ‘qubits'. A qubit is a two-level system that exhibits quantum properties: superposition and entanglement. Superposition refers to the ability that a qubit has to not only reside in the state $| 0 \rangle $ or $| 1 \rangle $ like a classical bit, but also in the state

(1) \begin{equation*} |\psi\rangle = {\rm{cos}}(\theta /2)|0\rangle + {e^{i\varphi }}(\sin \theta )/2| 1 \rangle . \end{equation*}

**Figure 1.**
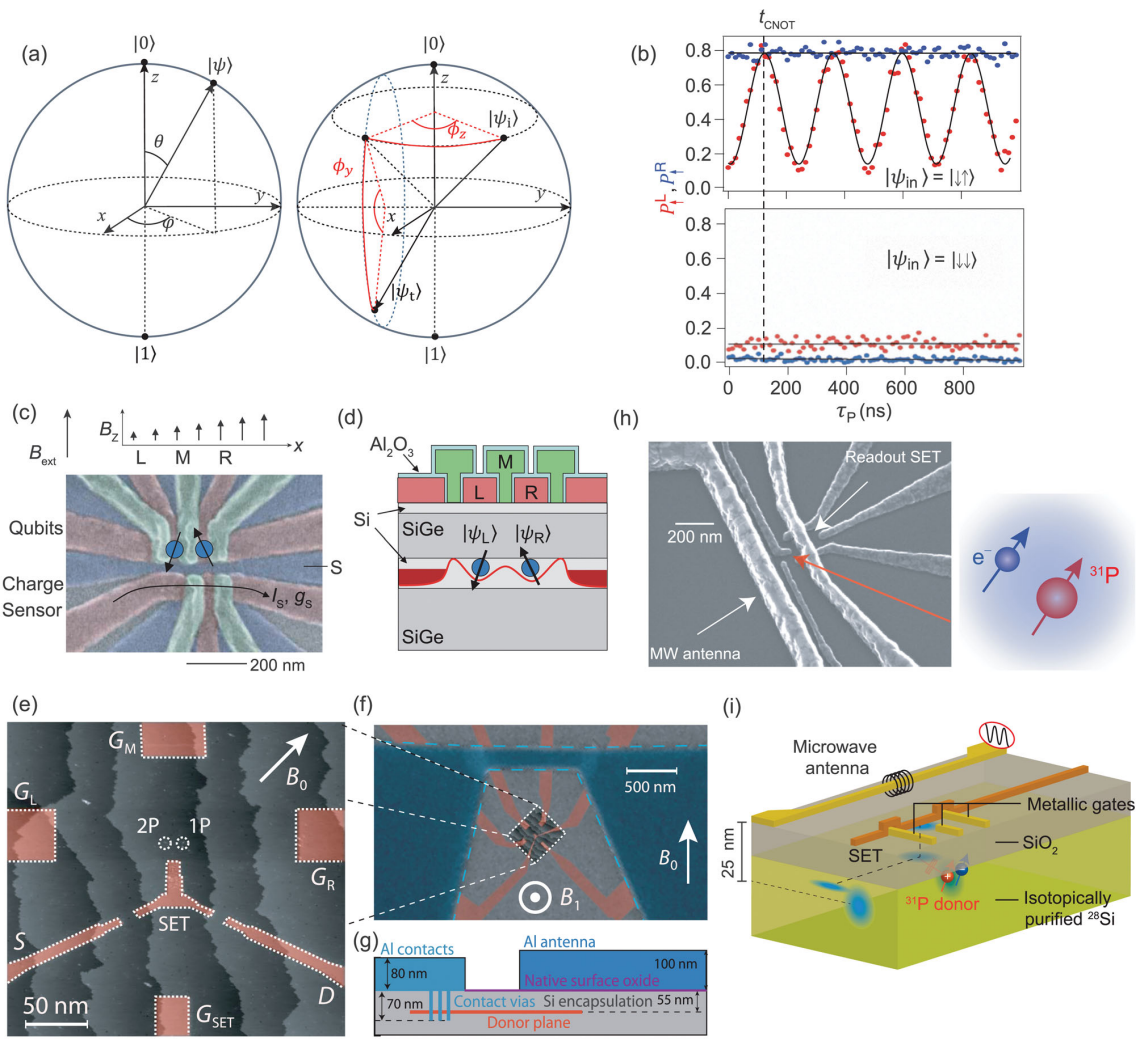
Single- and two-qubit gate control and devices for semiconductor qubits. (a) Bloch-sphere representation of a qubit. A superposition state $| \psi \rangle $ can be represented by a point on the sphere (left). An arbitrary rotation from the initial state $| {{\psi _i}} \rangle $ to the target state $| {{\psi _t}} \rangle $ can be decomposed by successive rotations about the *z* and *y* axes for *φ_z_* and *φ_y_*, respectively (right). (b) The spin-up probability of the spin-up state for the right qubit $P_ \uparrow ^{\rm{R}}$ (blue) and the left qubit $P_ \uparrow ^{\rm{L}}$ (red) as a function of interaction time ${\tau _{\rm{p}}}$ for input states $| { \downarrow \uparrow } \rangle $ and $| { \downarrow \downarrow } \rangle $. The vertical dashed line at ${\tau _{\rm{p}}} = 130\,\,{\rm{ns}}$ corresponds to a CNOT gate. (Adapted from [17].) (c) and (d) are a false-color SEM image and a schematic cross-section of a Si/SiGe DQD, respectively. The DQD with two electrons confined in the potential created by gates L, M and R is used to form two spin-1/2 qubits and a SET under the DQD is used to work as a charge sensor. A slanting Zeeman field was created by a micro-magnet (not shown) for qubit control. (Adapted from [17].) (e), (f) and (g) are images and schematics for the device fabricated by STM hydrogen lithography. (Adapted from [30].) (e) Large-scale STM image of the device; red areas are P-doped to form a SET, source and drain leads, and electrostatic gates. A donor molecule (2P) and single donor (1P) are shown by two circles. (f) False-color composite SEM and STM image showing the buried donor structures (red) and the aluminum antenna (blue). (g) Vertical cross-section of the donor device, showing the thicknesses (not to scale) and relative positions of the silicon, phosphorus, oxide and aluminum layers. (h) and (i) are a SEM image and schematic oblique view of a device fabricated by ion implantation, highlighting the position of the P donor, the MW antenna and the readout SET. (Adapted from [31] and [51].)

Here *θ* and ϕ are real numbers that define a point on a unit 3D sphere. Thus an arbitrary qubit state can be described as a point on the surface of a sphere, as depicted in Fig. 1a, which is termed a Bloch sphere. The basis states $| 0 \rangle $ and $| 1 \rangle $ are the north and south poles of the sphere, respectively, while the two superposition states $1/\sqrt 2 (| 0 \rangle + | 1 \rangle )$ and $1/\sqrt 2 (| 0 \rangle - | 1 \rangle )$ are on the equator.

The property of entanglement describes the correlation of different qubits during processing, i.e. a two-qubit state can be $1/\sqrt 2 (|01\rangle + |10),$ in which one qubit state depends on the other: if the first qubit were in state $| 0 \rangle $, the other qubit would be in state $| 1 \rangle $, and vice versa. By taking advantage of these two significant properties, many quantum algorithms have been proposed to give a nearly exponential speed-up compared to classical computing for a variety of problems, such as prime factorization [11], data searching [12], numerical optimization [13], chemical simulation [14], machine learning [15], etc. Since these problems are very common in the fields of banking, Internet, business, industry and scientific research, these quantum algorithms are believed to have a widespread use in the future.

All the quantum algorithms are based on a certain quantum computing model, varying from the quantum circuit, one-way quantum computation, adiabatic quantum computation and topological quantum computation. These four models are equivalent in computational power; among them, the quantum circuit model is most frequently used. In the circuit model, it has been proved that arbitrary single-qubit rotations plus two-qubit controlled-NOT gates are universal, i.e. they can provide a set of gates to implement any quantum algorithm [16]. As Fig. 1a shows, for a certain initial state $| {{\psi _i}} \rangle $ on the Bloch sphere, an arbitrary target state $| {{\psi _t}} \rangle $ can be achieved just by successive rotations about the *z* and *y* axes for *φ_z_* and *φ_y_*, respectively. In fact, as long as one can control rotations around two different axes of the Bloch sphere, arbitrary single-qubit rotations can be performed; this is also known as universal single-qubit control. On the other hand, a two-qubit controlled-NOT (CNOT) gate implies that one qubit state can be controlled by another. It acts on two qubits and a π rotation around the *x* axis is performed on the target qubit only when the control qubit state is $| 1 \rangle $. This intriguing phenomenon is shown in Fig. 1b, an experimental result from Zajac *et al.* [17], in which the ground state $| 0 \rangle $ ($| 1 \rangle $) is denoted by spin-down ${|}\!\! \downarrow \rangle $ (spin-up ${|}\!\! \uparrow \rangle $). In this figure, as manifested by the spin-up probabilities, the left qubit (red) shows rotations around the *x* axis as a function of interaction time when the right qubit (blue) is initialized in $| 1 \rangle $, whereas it keeps its initial state all the time when the right qubit is initialized oppositely. The vertical dashed line at which the two left qubit states are exactly opposite corresponds to a CNOT gate. Therefore, the core issue of building a quantum computer is to prepare a qubit with high-fidelity single- and two-qubit gates. The control fidelity depends on two factors: the coherence time and the manipulation time. Coherence time, also called dephasing time, is usually termed *T*_2_ and indicates how long a qubit can keep its quantum properties, while manipulation time, characterized by a rotation angle of π (*T*_π_) or 2π (*T*_2π_), refers to the time required for a single manipulation. In qubit experiments, the coherence time can be obtained by measuring the decay time of Larmor precession and Ramsey fringes [10]. Due to the instrumental imperfections, these decay times are usually smaller than *T*_2_ and are termed *T*_2_*. To get rid of these imperfections, dynamical decoupling pulses can be utilized and the resulting decay time is the intrinsic *T*_2_. In some experiments when these two parameters cannot be obtained, the decay times of other coherent oscillations are also used to estimate the qubit coherence, such as the decay time of Rabi oscillations (*T*^Rabi^). Usually, the Rabi decay time *T*^Rabi^ is longer than *T*_2_ since the concatenation of its oscillations plays a similar role to dynamical decoupling and during half of its time the qubit stays in the eigenstate that is less affected by the dephasing effect. One application of *T*^Rabi^ is the proposed quality factor *Q* = *T*^Rabi^/*T*_π_ [18] to characterize the qubit fidelity. A rough estimate of the qubit fidelity via *Q* is that *Q* ∼ 100 suggests a fidelity above 99% and *Q* ∼ 1000 suggests a fidelity above 99.9%. In the fields lacking fidelity measurements, the *Q* value is often used as a reference. Nevertheless, no matter how high the fidelity is, small errors can still be propagated and amplified through successive manipulations until the computation process is destroyed. To tackle this problem, a solution is to build a fault-tolerant quantum computer with qubits encoded by error-correcting codes. An example of these codes is the surface code, which requires a 2D array of qubits with single- and two-qubit gate fidelities above the threshold of 99% [19]. If qubits can be prepared meeting this requirement, millions of qubits encoded by surface code can be employed for running effective quantum algorithms.

In 1998, Loss and DiVincenzo [20] first proposed to utilize semiconductor quantum dots for manipulating single spins as qubits. A typical device of gate-defined lateral quantum dots is shown in Fig. 1c and d; the electrodes on the surface of the Si/SiGe heterostructure can form quantum potentials in the Si well to trap electrons, and the electron spins can be manipulated as qubits when an external magnetic field is applied. The upper half of Fig. 1c is a double quantum dot (DQD) to form two spin-1/2 qubits and the lower half is a single quantum dot (SQD) acting as a charge sensor to measure the charge states of the DQD, which is also called a single electron transistor (SET). In fact, quantum dots can be formed in various systems, including GaAs/AlGaAs heterostructures [21], silicon metal–oxide–semiconductor (MOS) and silicon-on-insulator (SOI) [22], nanowires [23], nanotubes [24], graphene [25], van der Waals heterostructures [26,27], and self-assembled crystals [28]. It is worth mentioning that quantum dots based on Si/SiO_2_ and SOI technology are both CMOS compatible and in this article we denote the former as silicon MOS and the latter as SOI for clarity. On the heels of the proposal for quantum-dot-based electron spins, Bruce Kane [29] showed that the nuclear spin of a single ^31^P donor in silicon can also be controlled as a qubit. There are two approaches to fabricate the device: scanning tunneling microscopy (STM) hydrogen lithography and ion implantation. For the former approach, the STM tip enables atomic-scale precision of placing P atoms in silicon. Figure 1e is an STM image of a device fabricated using this approach, showing a single donor (1P) and a donor molecule (2P) in the center for spin manipulation and beneath them is a SET for charge sensing. The blue area in Fig. 1f is an aluminum antenna generating an oscillating magnetic field over the device, and Fig. 1g is the vertical cross-section showing the relative position of the antenna and the silicon device [30]. For the latter approach, P ions are implanted into a very small region of the silicon using mask resists. Figure 1g and h show a scanning electron microscopy (SEM) image and the schematic of a device fabricated by ion implantation. In Fig. 1g, a P donor was implanted in the area denoted by the red arrow, and the spins of both the electron bound to the donor and the donor nucleus can be used as qubits [31]. Also, the SET and the Al antenna are used for readout and manipulation. In 2003, Hayashi and co-workers also investigated the coherent manipulation of electronic states of a DQD in the GaAs/AlGaAs heterostructure and showed the opportunity to implement a charge qubit [32] in a semiconductor DQD. These proposals together resulted in a subsequent firestorm of experimental activities [7]. So far, single- and two-qubit gate control has been achieved with fidelity above 99.9% [18,33] and 98% [34] respectively, approaching the surface code threshold for fault-tolerant computing. Also, thanks to the advanced semiconductor technology, several proposals taking advantage of today's semiconductor processing tools to scale up to 2D grids [35–39] have been put forward. Therefore, it is believed that there is a huge opportunity to realize a scalable fault-tolerant semiconductor quantum computer in the future.

In the following, we will begin with discussing single-qubit control for different types of semiconductor qubits and then move to two-qubit gates. Then, the challenges and also the opportunities for building a quantum computer will be discussed. Finally, we will introduce the views on semiconductor quantum computation around the world and anticipate that the research on semiconductor quantum devices may have a great influence in the following years.

## SINGLE-QUBIT GATE IN SEMICONDUCTOR

As discussed in the introduction, both the spin and charge degrees of the electrons and donor nucleus can be employed as qubits [7,40]. For the spin degree, spin-1/2 qubits, singlet–triplet qubits and exchange-only qubits have been proposed and realized in experiments successively. To take advantage of both spin and charge degrees, the hybrid qubit has also been presented as a competitive candidate. In this section, we restrict our scope to the single-qubit control of these qubits, and will discuss the two-qubit gate in the next section.

### Spin-1/2 qubit

Once an electron or nucleus is put into a magnetic field *B*_0_, the energy levels of spin-up and spin-down are no longer degenerate and split by the so-called Zeeman energy. This is a two-level system that can be used as a qubit and we call it a spin-1/2 qubit to distinguish it from other types of spin qubits. To manipulate this type of qubit, microwave (MW) bursts via an antenna were used to generate an oscillating magnetic field [30,31], as illustrated in Fig. 1f and h. This approach is called electron spin resonance (ESR) for controlling electron spins or nuclear magnetic resonance (NMR) for controlling nuclear spins. In the rotating frame, the control Hamiltonian can be written as:

(2) \begin{eqnarray*} {\bf{H}}_{\boldsymbol R} & \approx & \left( { - {\omega _0} + {\boldsymbol{\omega }}} \right){\sigma _z}/2 \nonumber\\ &&- {\omega _R}[\cos \left( \phi \right){\sigma _x}/2 - \sin \phi \,\,{\sigma _y}/2].\nonumber\\ \end{eqnarray*}

For simplicity, we use natural units throughout the article. Here, **ω**, ω_0_, ω_R_ are MW frequency, Larmor frequency and Rabi frequency, respectively. The latter two satisfy the condition ${\omega _{\rm{R}}} = {\rm{\gamma }}| {{{{\bf B}}_{\rm{0}}}} |$ and ${\omega _{\rm{0}}} = {\rm{\gamma }}| {{{{\bf B}}_{\rm{1}}}} |$ with γ the gyromagnetic ratio, ***B***_0_ the external magnetic field and ***B***_1_ the oscillating magnetic field perpendicular to ***B***_0_. Thus the Larmor frequency corresponds to the Zeeman energy splitting of the qubit. ***B***_1_ is produced by the MW antenna and its magnitude is directly proportional to the current through the antenna. The Pauli matrices ${\sigma _z}$, ${\sigma _x}$ and ${\sigma _y}$ suggest rotations around the *z* axis, *x* axis and *y* axis of the Bloch sphere, respectively. Therefore, a sequence of MW bursts with a frequency ${\boldsymbol{\omega }} = {\omega _0}$ on resonance with the qubit and initial phase $\phi = 0$ will drive the qubit to rotate around the *x* axis. In particular, the nutation between $| 0 \rangle $ and $| 1 \rangle $ is usually called Rabi oscillation. When the MW is halted for a time, or the relative phase of successive MW bursts is varied, the qubit will acquire a rotation angle around the *z* axis. Universal single spin control can thus be achieved using this approach. Alternatively, another approach to manipulate the spin-1/2 qubit is electric-dipole spin resonance (EDSR). In this approach, a magnetic field gradient is applied with the help of spin–orbital coupling (SOC) of the semiconductor or an integrated micro-magnet, and the electron in this environment can feel an effective oscillating magnetic field if it is driven by an oscillating electric field. Therefore, MW bursts can be applied directly on a single electrode and ***B***_1_ is proportional to its voltage amplitude. One example using this approach is shown in Fig. 1c; there is a magnetic field gradient in the device generated by an integrated micro-magnet (not shown), and the MW bursts are applied on gate S for qubit control [17].

**Figure 2.**
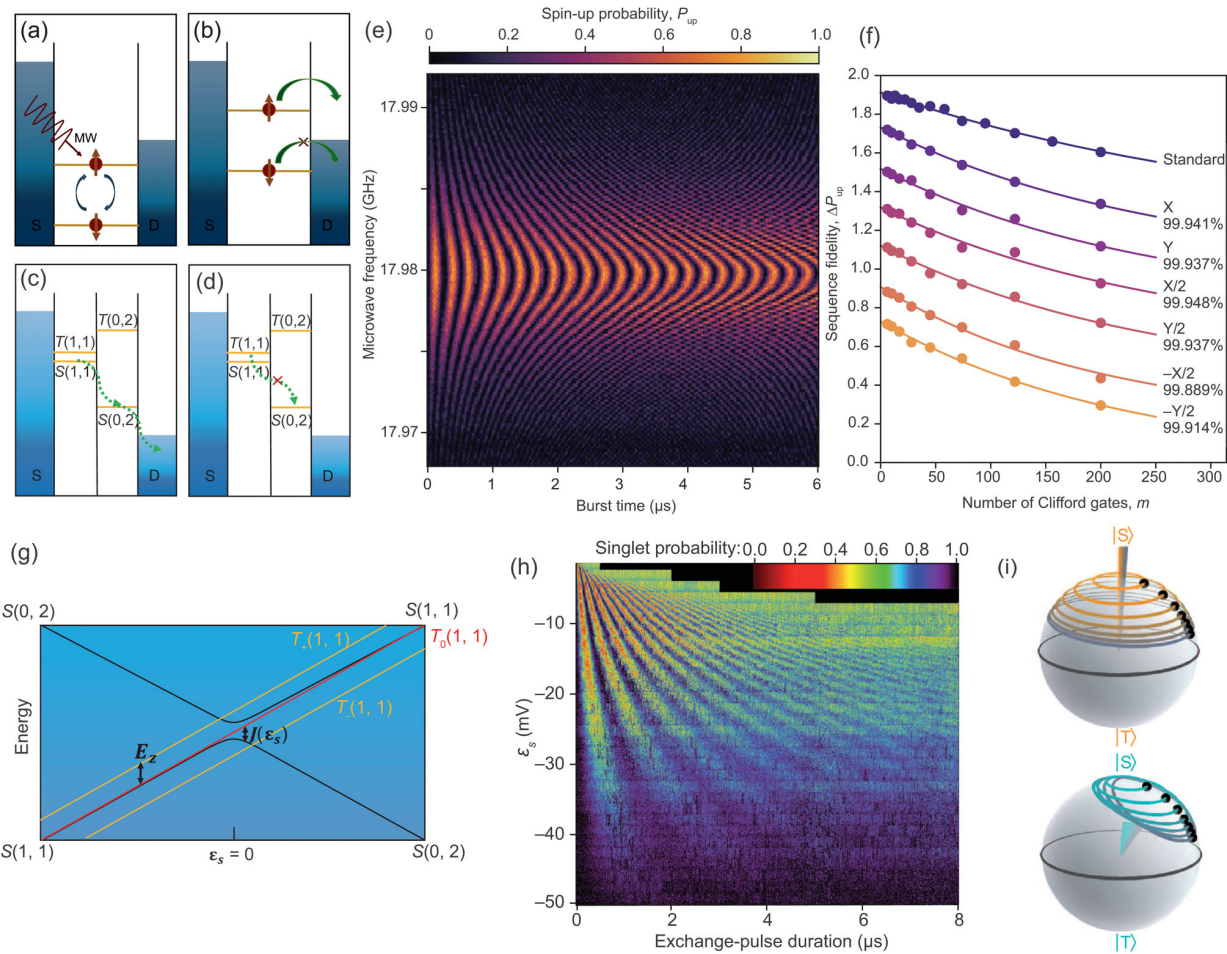
Spin-1/2 qubit and singlet–triplet qubit. (a) and (b) are diagrams showing the process of control and readout based on spin-selective tunneling. (a) At the stage for qubit control, both energy levels of spin-up and spin-down are under the Fermi level of drain. (b) At the stage for readout, the energy levels in the dot are tuned so that the Fermi level of drain is between the energy levels of spin-down and spin-up. (c) and (d) are diagrams showing the phenomenon of spin blockade: *S*(1, 1) can move to *S*(0, 2) while *T*(1, 1) cannot. (e) The probability of spin-up *P*_up_ as a function of MW burst time and frequency detuning. (Adapted from [17,18].) (f) Sequence fidelities for standard (topmost) and interleaved randomized benchmarking (annotated in the figure along with extracted fidelities). Traces are offset by an increment of 0.2 for clarity. Visibilities are within 0.72 ± 0.012. (Adapted from [17,18].) (g) Energy-level spectrum of two spin states in a DQD as a function of detuning ${\varepsilon _s}$. A magnetic field splits the triplet states by the Zeeman energy ***E_z_*** and the exchange interaction splits *S* and *T*_0_ by $J({\varepsilon _s})$. (h) Singlet probability as a function of exchange-pulse duration and detuning ${\varepsilon _s}$. (Adapted from [68].) (i) Bloch-sphere representations of state evolution in the case $J({\varepsilon _s}) > \Delta $***E_z_*** (top) and $J({\varepsilon _s}) < \Delta $***E_z_*** (bottom). (Adapted from [68].)

Readout of the spin-1/2 qubits relies on a spin–charge conversion as spin-selective tunneling [41–43] or spin blockade [44,45], and after the conversion the charge signal is detected by a nearby charge sensor. The procedure for spin-selective tunneling is illustrated in Fig. 2a and b, when a spin-1/2 qubit is under MW control, the energy levels of both spin states are under the Fermi level of the drain, and, after control, the energy levels in the quantum dot are tuned so that the energy level of spin-up is higher than the Fermi level of the drain and spin-down is lower. In this energy-level alignment, only the electron with spin-up can tunnel out of the quantum dot and thus the spin state can be distinguished by observing the electron tunneling signal. This approach was first demonstrated by Elzerman *et al.* in 2004 [41], and they achieved single-shot readout of a single electron spin for the first time. An adaptation of Elzerman's method is to use the tunneling rate difference instead of the energy difference of two energy levels to differentiate spin states; this was first demonstrated by Hanson *et al.* in 2005 [42]. Here we term the former energy-selective readout and the latter tunnel-rate-selective readout. Inspired by Elzerman *et al.*'s work, in 2010, Morello *et al.* demonstrated the first single-shot spin readout of an electron bound to a donor in silicon, in which they used the electrochemical potential of the SET to play the role of the Fermi level of a drain for energy selection [43]. As for the spin blockade, it utilizes another spin as an ancilla qubit to read the spin state in the singlet–triplet basis. There are four basis states for two spins in a magnetic field and they can be sorted into a singlet and three triplets:

(3) \begin{eqnarray*} S &=& 1/\sqrt 2 ({|}\!\! \uparrow \downarrow \rangle - | { \downarrow \uparrow } \rangle ),\nonumber\\ {T_0} &=& 1/\sqrt 2 \ (| { \uparrow \downarrow }\rangle \nonumber\\ && +\, |{ \downarrow \uparrow } \rangle ),\ \ {T_ + } = | { \uparrow \uparrow } \rangle,\nonumber\\ {T_ - } &=& \ |{ \downarrow \downarrow } \rangle . \end{eqnarray*}

Here, *S* and *T*_0_ are separated by the exchange interaction strength *J*, and the three triplets are split by the Zeeman energy ***E**_**z**_*. We denote the singlet with each electron occupying one quantum dot as *S*(1, 1) and the one with two electrons both occupying the right dot as *S*(0, 2). This type of notation also applies to the triplets. If we suppose that both spins are initialized in $| { \downarrow \downarrow } \rangle $, the MW bursts on the left spin will lead the two spin states to oscillate between $| { \downarrow \downarrow } \rangle $ and $| { \uparrow \downarrow } \rangle $. In experiments, $| { \downarrow \downarrow } \rangle $ is usually mapped to *T*_(1, 1) and $| { \uparrow \downarrow } \rangle $ is mapped to *S*(1, 1). As illustrated in Fig. 2c and d, only the *S*(1, 1) state can transit to *S*(0, 2) and other states are prohibited because of spin blockade. Thus a nearby charge sensor that can differentiate charge states (1, 1) and (0, 2) is able to read out the spin state. For simplicity, in the figure we use *T*(1, 1) for those triplets. This measurement method was first demonstrated by Petta *et al.* in 2005, and with mapping $| { \uparrow \downarrow } \rangle $ to *S* and $| { \downarrow \uparrow } \rangle $ to *T*_0_, they implemented a controlled two-qubit gate exchanging the spin direction [44]. Then, combined with MW bursts, Koppens *et al.* demonstrated the first driven coherent single spin rotations in 2006 [45]. Furthermore, the visibility of readout in the singlet–triplet basis can be enhanced by charge-state latching [46,47] and intermediate excited states [48]. In Harvey-Collard *et al.*'s work, they achieved a measurement fidelity as high as 99.86% via charge-state latching, and in 2018, Fogarty *et al.* demonstrated measuring a spin-1/2 qubit using this new method [49].

When Petta *et al.* and Koppens *et al.* first demonstrated two- and single-qubit gates respectively in GaAs quantum dots, they met a serious problem: that the hyperfine interactions (HI) with the GaAs host nuclei have a non-trivial influence on the coherence of the spin-1/2 qubit and limit its dephasing time *T*_2_* only to tens of nanoseconds [50]. An alternative approach is to use group IV host materials, Si or Ge, to eliminate the random nuclear spins [51]. In 2014, Kawakami *et al.* demonstrated a spin-1/2 qubit with *T*_2_* ∼ 1 μs in natural silicon [52]. Still, the residual ^29^Si nuclear spins in natural silicon can worsen the dephasing time, and to overcome this, Veldhorst *et al.* performed a similar experiment in purified silicon with ^29^Si down to 800 ppm. The great reduction of ^29^Si enabled them to measure a *T*_2_* as long as 120 μs and obtained a fault-tolerant fidelity of 99.6% [53], which is at the threshold of surface code for fault-tolerant quantum computing. However, limited by ESR heating, the manipulation time of their device is up to *T*_π_ ∼ 1 μs. In 2017, by using EDSR rather than ESR, Yoneda *et al.* [18] reduced the manipulation time *T*_π_ to ∼17 ns and, keeping *T*_2_* ∼ 20 μs, they reported a control fidelity over 99.9%. The high-frequency Rabi oscillation with *T*_π_ ∼ 128 ns and the randomized benchmarking of the control fidelity for different types of gates are shown in Fig. 2e and f, respectively. As for donors in silicon, in 2014, Muhonen *et al.* demonstrated a dephasing time as long as 600 ms for ^31^P^+^ spin and reached a control fidelity >99.99%. Moreover, the intrinsic dephasing time measured by dynamical decoupling pulses resulted in *T*_2_ exceeding 30 seconds, implying a future application in storing quantum information. For the electron spin bound to the donor, they also obtained *T*_2_* ∼ 270 μs and a fidelity over 99% [31].

Except for electrons and nuclei, hole spins can also be encoded as spin-1/2 qubits. The small HI and strong SOC of the hole spin promises both a long dephasing time and a short manipulation time; therefore, this field has become very active since the first measurement of the coherence time of a single hole spin in an InGaAs quantum dot [54]. So far, hole-spin qubits have been demonstrated in an SOI DQD [55] and a Ge hut wire DQD [56]. With strong SOC, the best manipulation rate can be over 140 MHz, i.e. the rotation time *T*_2π_ can be as short as ∼7 ns. However, the measured dephasing times were both at least one order of magnitude lower than that of the electron spins, and the qubit control cannot be proved at a single hole level. Other approaches include manipulating spins of holes that are bound to acceptors in silicon [57] or that are trapped in quantum dots fabricated from the p-GaAs/AlGaAs heterostructure [58], the silicon MOS [59], Ge/GeSi heterostructures [60] and core–shell nanowires [61].

### Singlet–triplet qubit

Another type of spin qubit is encoded by two eigenstates of two spins [62]. Usually, the encoded states are *S* and *T*_0_, and we thus call it singlet–triplet qubit. The effective control Hamiltonian can be written as follows:

(4) \begin{equation*} {{\boldsymbol{H}}_{ST}} = J({\varepsilon _s}){\sigma _z}/2 + \Delta {{\boldsymbol{E}}_{\boldsymbol{z}}}{\sigma _x}/2. \end{equation*}

Here, $J({\varepsilon _s})$ is the energy of exchange splitting of *S* and *T*_0_, where the detuning ${\varepsilon _s}$ denotes the electrochemical potential difference of different charge occupation states, and $\Delta {\boldsymbol E}_{\boldsymbol z}$ is the Zeeman energy difference of two spins, which may be caused by different *g*-factors [63], i.e. $\Delta {{\boldsymbol{E}}_z} = \Delta g{\mu _B}{{\boldsymbol{B}}_z}$, or magnetic field gradients [64,65], i.e. $\Delta {{\boldsymbol{E}}_{\boldsymbol{z}}} = g{\mu _B}\Delta {{\boldsymbol{B}}_{\boldsymbol{z}}}$. As shown in Fig. 2g, when the detuning point is set negative and far away from zero, $J({\varepsilon _s})$ will be vanishing and thus the qubit will rotate around the *x* axis; in contrast, when the detuning point is tuned in the positive direction until $J({\varepsilon _s}) > > \Delta {{\boldsymbol{E}}_{\boldsymbol{z}}}$, the qubit will rotate around the *z* axis. In this control procedure, only the parameter ${\varepsilon _s}$ is used and thus the need for ESR or EDSR is removed compared to spin-1/2 qubits. After manipulation, the qubit can be measured directly using spin blockade. The singlet–triplet qubit was first demonstrated experimentally in GaAs quantum dots with *T*_2_* ∼ 10 ns and a rotation period *T*_2π_ ∼ 720 ps around the *z* axis by Petta *et al.* in 2005 [44]. The dephasing time is mainly limited by the fluctuating nuclear field and a lot of subsequent research concentrated on how to improve it. By using dynamical nuclear polarization (DNP) for controlling the nuclear field in a feedback loop, in 2010, Bluhm *et al.* demonstrated *T*_2_* ∼ 94 ns [66], and, further, with dynamical decoupling pulses to filter low-frequency noises, they achieved an intrinsic dephasing time *T*_2_ exceeding 200 μs [67]. Similar to spin-1/2 qubits, silicon was also expected to replace GaAs as a host material for singlet–triplet qubits. In 2012, Maune *et al.* first demonstrated a singlet–triplet qubit in a Si/SiGe DQD, reporting *T*_2_* ∼ 360 ns [68]. The *S*–*T*_0_ oscillations in this experiment can be observed in Fig. 2h. Figure 2i shows the Bloch-sphere representations of state evolution in the case $J({\varepsilon _s}) > {\boldsymbol{\Delta }}{{\boldsymbol{E}}_{\boldsymbol{z}}}$ and $J({\varepsilon _s}) < {\boldsymbol{\Delta }}{{\boldsymbol{E}}_{\boldsymbol{z}}}$. However, the spin blockade can be lifted easily due to the small splitting of the two low-lying valley states in the Si/SiGe heterostructure, which puts a great hurdle in front of the reproducibility of singlet–triplet qubits in this material. In 2014, Shulman *et al.* found that a real-time Hamiltonian estimation (RHE) could be used to suppress qubit dephasing and they measured *T*_2_* more than 2 μs in a GaAs DQD, even one order of magnitude over that in silicon [69]. In 2016, Malinowski *et al.* improved the design of dynamical decoupling sequences to use it as a notch filter for nuclear noises and improved *T*_2_ to 870 μs [70]. As for charge noises, in 2017, Nichol *et al.* [71] discovered that a large $\Delta {\boldsymbol E}_{\boldsymbol z}$ could suppress charge noises and combined it with RHE; they reported a record single-qubit gate fidelity of ∼99% in GaAs.

Apart from these, singlet–triplet qubits can also be encoded by *S* and *T*_+_ [72], or implemented in other systems, such as donors in silicon [73,74] and hybrid donor–dot architecture [75]. It is noteworthy that for donors in silicon, the transitions between (1, 1) and (0, 2) are harder to distinguish for the special charge sensor arrangement and thus the energy-selective readout or tunnel-rate-selective readout like spin-1/2 qubits are preferred. These two readout methods for singlet–triplet qubits have been investigated by Broome *et al.* [74] and Dehollain *et al.* [73], respectively. For the hybrid donor–dot singlet–triplet qubits, the readout relies on the aforementioned latching-enhanced spin blockade.

### Exchange-only qubit

**Figure 3.**
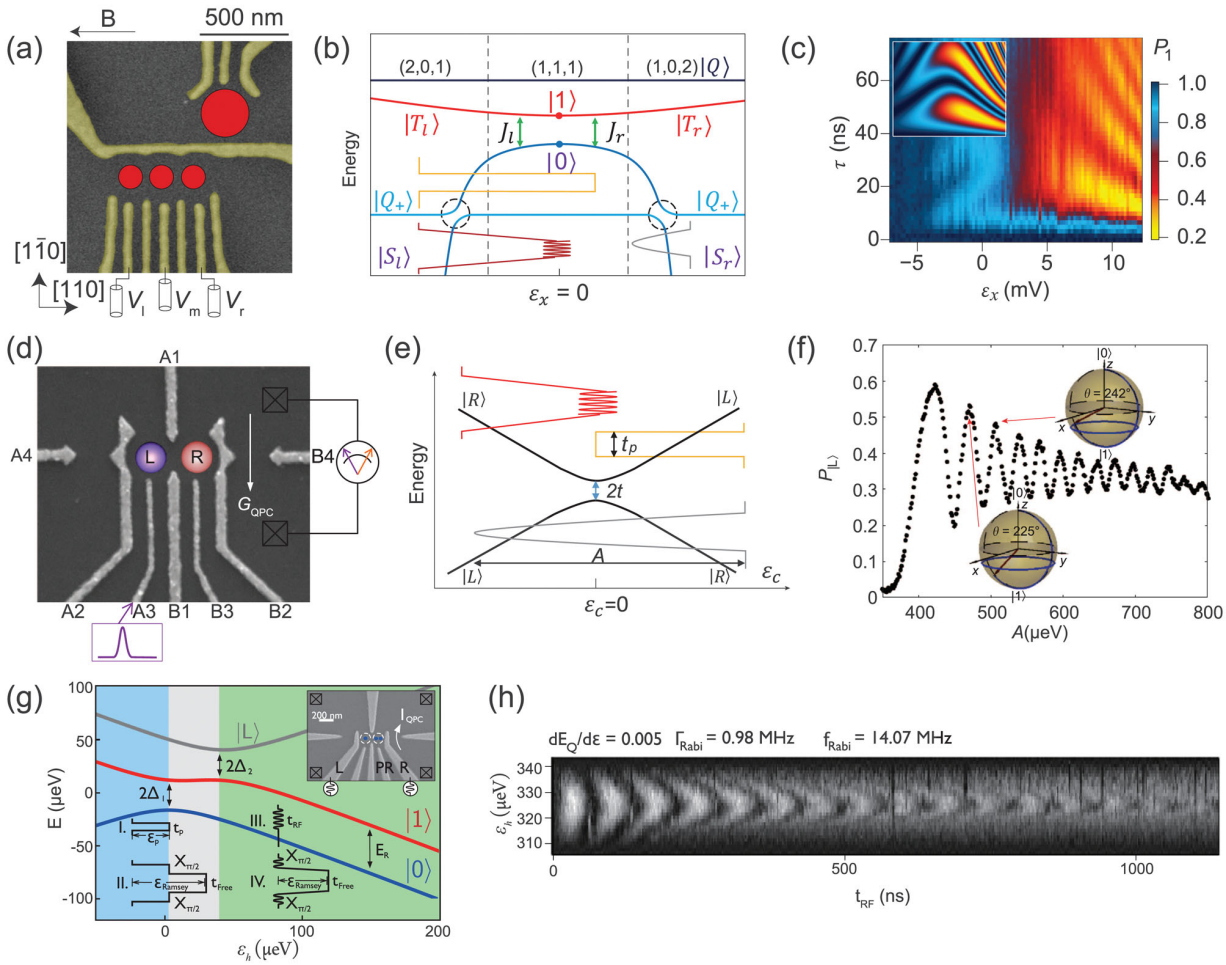
Implementations of the exchange-only qubit, the charge qubit and the hybrid qubit. (a) False-color SEM image of a TQD device for an exchange-only qubit, with a SET on the top for charge sensing. (Adapted from [78].) (b) Energy levels as a function of detuning ${\varepsilon _x}$ for the exchange-only qubit. Two anti-crossings are shown by two dotted circles. Yellow, red and gray pulse shapes are shown to denote the relative positions of detuning for Larmor oscillation, Rabi oscillation and LZS interferences. (c) The probability *P*_1_ of detecting the state ${|{S_l}} \rangle $ as a function of pulse position ${\varepsilon _x}$ and wait time τ. Inset is a simulation result of qubit evolution as a function of exchange without noise. (Adapted from [78].) (d) A SEM image of a DQD device for a charge qubit with two QPCs for readout. ${|L} \rangle $ and ${|R} \rangle $ are denoted by two circles in the DQD. (Adapted from [88].) (e) Energy levels of a charge qubit as a function of detuning ${\varepsilon _c}$. Red, yellow and gray pulse shapes are shown to denote the relative positions of detuning for Rabi oscillation, Larmor oscillations and LZS interferences. (f) Charge-state probability ${|{P_L}} \rangle $ as a function of LZS pulse amplitude *A*. The Bloch-sphere representations for two interference nodes are also labeled. (Adapted from [88].) (g) Energy spectrum as a function of ${\varepsilon _h}$ and pulse sequences for the hybrid qubit. Inset is a SEM image of a DQD device with a QPC for readout. (Adapted from [94].) (h) Rabi oscillations demonstrating a decay time longer than 1 μs. (Adapted from [94].)

Though singlet–triplet qubits can be driven all electrically, they still need a Zeeman energy difference to achieve universal single-qubit control. How about implementing a qubit solely by exchange interaction? This idea leads to the exchange-only qubit [76]. As illustrated in Fig. 3a, this type of qubit is composed of three electrons in a triple quantum dot (TQD) [77–79]. There are eight basis states for three spins, and among them $| {{S_l}} \rangle = \ 1/\sqrt 2 (| { \uparrow \downarrow \uparrow } \rangle - {\rm{|}} { \downarrow \uparrow \uparrow } \rangle )$ and $| {{T_l}} \rangle = \ 1/\sqrt 6 (| { { \downarrow \uparrow \uparrow } \rangle + } | { \uparrow \downarrow \uparrow } \rangle - 2{\rm{|}} { \uparrow \uparrow \downarrow } \rangle {\rm{)}}$ are separated by exchange splitting ${J_l}({\varepsilon _x})$; $| {{S_r}} \rangle = \ 1/\sqrt 2 (| { \uparrow \uparrow \downarrow } \rangle - {\rm{|}} { \uparrow \downarrow \uparrow } \rangle {\rm{)}}$, and $| {{T_r}} \rangle = 1/ \sqrt 6 (| { { \uparrow \uparrow \downarrow } \rangle + } | { \uparrow \downarrow \uparrow } \rangle - 2{\rm{|}} { \downarrow \uparrow \uparrow } \rangle {\rm{)}}$ are separated by ${J_r}({\varepsilon _x})$. Here, $| {{S_l}} \rangle (| {{S_r}} \rangle )$ and $| {{T_l}} \rangle (| {{T_r}} \rangle )$ are singlet-like state and triplet like states, respectively, which can be inferred from the state of the left (right) two spins. The two exchange-splitting energies ${J_l}({\varepsilon _x})$ and ${J_r}({\varepsilon _x})$ are associated with the left pair and the right pair of quantum dots, respectively, and the detuning ${\varepsilon _x}$ denotes the relative electrochemical potential of the charge configurations (2, 0, 1), (1, 1, 1) and (1, 0, 2). As depicted in Fig. 3a, the ground state $| 0 \rangle = 1/\sqrt 6 (| { { \uparrow \uparrow \downarrow } \rangle + } | { \downarrow \uparrow \uparrow } \rangle - 2{\rm{|}} { \uparrow \downarrow \uparrow } \rangle {\rm{)}}$ and the excited state $| 1 \rangle = 1/\sqrt 2 (| { \uparrow \uparrow \downarrow } \rangle - {\rm{|}} { \downarrow \uparrow \uparrow } \rangle {\rm{)}}$ are encoded in the center of the (1, 1, 1) charge configuration with ${J_l}({\varepsilon _x}) = {J_r}({\varepsilon _x})$. Also, there are two extra states $| Q \rangle = 1/\sqrt 3 (| { { \uparrow \uparrow \downarrow } \rangle + } | { \uparrow \downarrow \uparrow } \rangle - 2{\rm{|}} { \downarrow \uparrow \uparrow } \rangle {\rm{)}}$ and $| {{Q_ + }} \rangle = {{|}\!\! \uparrow \uparrow \uparrow } \rangle $ in the energy-level spectrum that may offer leakage channels when the qubit is under control. The control Hamiltonian can be described as:

(5) \begin{equation*} {{\boldsymbol{H}}_{EX}} = - {J_l}\left( {{\varepsilon _x}} \right){\sigma _l}/2 - {J_r}\left( {{\varepsilon _x}} \right){\sigma _r}/2, \end{equation*}

in which

(6) \begin{equation*} {\sigma _{l }}=\left( {{\sigma _z} - \sqrt 3 {\sigma _x}} \right)\!/2,\,\,{\sigma _{r }}=\left( {{\sigma _z} + \sqrt 3 {\sigma _x}} \right)\!/2. \end{equation*}

In the Bloch sphere, the axes ${\sigma _l}$ and ${\sigma _r}$ are 120° apart and thus universal single-qubit control can be achieved by directly tuning ${J_l}({\varepsilon _x})$ and ${J_r}({\varepsilon _x})$ via detuning pulses. This method is called Larmor precession and, as an example, a control pulse sequence (yellow) is drawn in Fig. 3b, indicating a detuning pulse from $| {{S_l}} \rangle $ to (1, 1, 1). After manipulation, the qubit state can be measured via spin blockade with $| 0 \rangle $ mapped to $| {{S_l}} \rangle (| {{S_r}} \rangle )$ and $| 1 \rangle $ mapped to $| {{T_l}} \rangle (| {{T_r}} \rangle )$. In 2010, Laird *et al.* first demonstrated an exchange-only qubit in a GaAs TQD with this approach [77], and then, in 2013, Medford *et al.* measured an inhomogeneous dephasing time *T*_2_* ∼ 25 ns and a rotation time *T*_2π_ as short as ∼21 ps [78]. The coherent oscillations in their experiment are shown in Fig. 3c. Another approach to control exchange-only qubits is to use Rabi oscillations. As the red pulse sequence in Fig. 3b shows, qubit manipulation can be implemented directly by applying MW bursts at zero detuning with a frequency in resonance with the energy gap between $| 0 \rangle $ and $| 1 \rangle $. The qubit controlled in this way is also called a resonant exchange qubit. In 2013, Medford *et al.* demonstrated a resonant exchange qubit and reported an intrinsic dephasing time *T*_2_ ∼ 19 μs and a rotation time *T*_2π_ ∼ 10 ns [79]. To make further improvements, other investigations also include reducing magnetic noise by performing experiments in silicon quantum dots [80] and suppressing charge noise by using MW bursts in a highly symmetric regime [81]. Moreover, the spin states of a TQD can also be controlled through Landau–Zener–Stückelberg (LZS) interferences. This was demonstrated by Gaudreau *et al.* in 2011 [82] and they encoded a qubit using the state $| 0 \rangle $ and $| {{Q_ + }} \rangle $. The hyperfine interaction that couples these two states results in two anti-crossings in the energy-level spectrum, which are denoted by dotted circles in Fig. 3b. An adiabatic pulse passing through one of the anti-crossings with an appropriate rise time can create a superposition state of $| 0 \rangle $ and $| {{Q_ + }} \rangle $ due to a Landau–Zener transition, and after a time the pulse goes across the anti-crossing again and back to its original position, resulting in LZS interferences. After that, a measurement in the basis of qubit eigenstates will show corresponding coherent oscillations. From the fit to the LZS model with their experimental results, they extracted a dephasing time *T*_2_* around 8–15 ns.

### Charge qubit

Besides the spin degree, quantum control of the charge states of an electron is also of interest. For a charge qubit, the ground state $| 0 \rangle $ and the excited state $| 1 \rangle $ can be defined by the excess electron occupation of a DQD, and as illustrated in Fig. d, they are usually denoted by $| R \rangle $ and $| L \rangle $, respectively. Readout of the qubit states can be implemented directly by a proximate charge sensor, a SET or a quantum point contact (QPC), or just the transport current from source to drain, so that it removes the need of any conversion like spin qubits. In Fig. 3d, the current through QPC is shown by the white arrow. The energy levels are depicted in Fig. 3e, with a Hamiltonian [32,83,84]:

(7) \begin{equation*} {{\boldsymbol{H}}_c} = {\varepsilon _c}{\sigma _z}/2 + t{\sigma _x}. \end{equation*}

Here ${\varepsilon _c}$ denotes the detuning energy between $| L \rangle $ and $| R \rangle $, and *t* is the inter-dot tunneling rate. As shown in Fig. 3e, a rectangular non-adiabatic voltage pulse (orange) that drives the qubit from the ground state $({\varepsilon _c} > 0)$ to the anti-crossing $({\varepsilon _c} = 0)$ can induce Larmor precession, resulting in a rotation around the *x* axis. If ${\varepsilon _c}$ is kept at the ground state, which is far away from the anti-crossing, the inter-dot tunneling rate will vanish, leading to a rotation around the *z* axis. Using this approach, the charge qubit was first demonstrated in GaAs quantum dots by Hayashi *et al.* in 2003, reporting a coherence time *T*_2_* ∼ 1 ns and a rotation time *T*_2π_ ∼ 435 ps [32]. Then the inhomogeneous dephasing time *T*_2_* was determined by Ramsey fringes as 60 ps [85]. Experiments based on Si/SiGe quantum DQDs were also reported with *T*_2_* ∼ 2.1 ns measured by Larmor oscillations, and *T*_2_* ∼ 127 ps and *T*_2_ ∼ 760 ps obtained by the Ramsey fringes and dynamical decoupling pulses, respectively [86]. Here, the flat band at the anti-crossing point makes the qubit less affected by the charge noise induced by detuning compared to the steeper point at ${\varepsilon _c} > 0$ and thus the coherence time of rotations around the *x* axis (Larmor oscillations) is much longer than that of the *z* axis (Ramsey fringes and dynamical decoupling pulses). Since the LZS interference is less sensitive to certain types of noise, the charge qubit was also investigated through LZS interference. In 2012, Stehlik *et al.* performed LZS interferometry of a semiconductor charge qubit via continuous microwave irradiation and observed the coherent oscillations of the qubit states [87]. In 2013, Cao *et al.* first observed the LZS interferences in the time domain and demonstrated an ultrafast universal qubit control with *T*_2π_ as short as ∼10 ps and intrinsic dephasing time *T*_2_ up to 4 ns that was extracted from the amplitude spectroscopy [88]. The adiabatic short pulse that they used to drive the qubit is shown in Fig. 3e; as described in the previous subsection, the LZS interference is finished after the pulse goes across the anti-crossing and back to its original position. The measured interferences as a function of pulse amplitude *A* are depicted in Fig. 3f, and, as shown by the Bloch spheres labeled at two interference nodes, the qubit is rotated around the *z* axis by 2π between every two successive interference fringes while the rotation angle of the *x* axis, *θ*, increases monotonically with pulse amplitude. Therefore, the qubit can be rotated around both the *x* and *z* axes within a single pulse and these rotations can be controlled arbitrarily by adjusting the pulse amplitude. Moreover, the charge qubit can also be controlled by applying resonant MW bursts at ${\varepsilon _c} = 0$ to induce Rabi oscillations and the two-axis control using MW bursts is just like that of spin-1/2 qubits and resonant exchange qubits. Here, the ground state and the excited state are changed to $| 0 \rangle \approx ( {| { L \rangle + } | R \rangle } )/\sqrt 2 $ and $| 1 \rangle \approx ( {| { L \rangle - } | R \rangle } )/\sqrt 2 $. In 2015, Kim *et al.* implemented a resonant charge qubit in a Si/SiGe quantum DQD, reporting *T*_2_* of 1.3 ns and *T*_2_ ∼ 2.2 ns [89]. With the improvement in coherence time, they measured an average qubit fidelity greater than 86%. In 2018, research on valley–orbit states in silicon also implied that the hybridized valley–orbit states can potentially be employed for higher fidelity control, where the energy band is flat with respect to a larger range of detuning [90].

### Hybrid qubit

Inspired by the fact that the coherence times of spin qubits are usually very long and the manipulation times of charge qubits are very short, one may question whether we can create a new type of qubit combining the advantages of both. An attempt originating from this idea is the hybrid qubit [91,92]. This type of qubit is encoded by two eigenstates of three electron spins in a DQD and was first demonstrated in a Si/SiGe heterostructure [93]. Figure 3g shows its energy levels as well as the device set-up [94]. The two lowest energy levels for qubit control are

(8) \begin{eqnarray*} |0 \rangle &=& |{\left. \downarrow \right\rangle _L}\ |{\left. S \right\rangle _R},\nonumber\\ | 1 \rangle &=& 1/\sqrt 3 |{\left. \downarrow \right\rangle _L}\ |{\left. {{T_0}} \right\rangle _R} - \sqrt {2/3} |{\left. \uparrow \right\rangle _L}|{\left. {{T_ - }} \right\rangle _R}.\nonumber\\ \end{eqnarray*}

The subscript *L* (*R*) denotes the spin state in the left (right) quantum dot, and the higher state $| {\rm{L}} \rangle $ in Fig. 3g is a primary leakage channel. On the basis of these three states, the Hamiltonian can be written as

(9) \begin{equation*} {{\boldsymbol{H}}_c} = \left( {\begin{array}{@{}*{3}{c}@{}} { - {\varepsilon _h}/2}&{{t_1}}&{{t_2}}\\ {{t_1}}&{ - {\varepsilon _h}/2}&0\\ {{t_2}}&0&{ - {\varepsilon _h}/2 + {E_R}} \end{array}} \right). \end{equation*}

Here, ${t_1}$ and ${t_2}$ are the tunnel couplings between $| 0 \rangle $ and $| 1 \rangle $, $| 1 \rangle $ and $| {\rm{L}} \rangle $, respectively, and ${\varepsilon _h}$ is the detuning between charge states (2, 1) and (1, 2), while ${E}_{R}$ is the energy separation between the two lowest valley–orbit states in the right dot. The energy-level spectrum can be divided into three regions: charge-like region (blue), hybrid region (gray) and spin-like region (green). In the spin-like region, ${E_R}$ is just the splitting energy of $| 0 \rangle $ and $| 1 \rangle $. The charge-like region can be used for readout using spin blockade with $|{ S \rangle _R}$ mapped to $| 0 \rangle $ and $|{ {{T_0}} \rangle _R}$ as well as $|{ {{T_ - }} \rangle _R}$ mapped to $| 1 \rangle $. In the readout regime, spin blockade will permit the transition between (1, 2) and (2, 1) for $|{ S \rangle _R}$ and prohibit it for $|{ {{T_0}} \rangle _R}$ and $|{ {{T_ - }} \rangle _R}$. Therefore, $| 0 \rangle $ and $| 1 \rangle $ can be distinguished from the charge occupation after the conversion. For qubit control, as shown by the pulses labeled (I)–(IV) in Fig. 3g, it can be performed either in the hybrid region by Larmor precession or in the spin-like region by Rabi oscillation. In the Larmor precession regime, a control pulse stops at ${\varepsilon _h} = 0$ and ${\varepsilon _h} > 0$ will rotate the qubit about the *x* and *z* axes, respectively. In 2014, Kim *et al.* measured a control fidelity of 85% for the *x* axis and 94% for the *z* axis using a Si/SiGe DQD [93]. This is only a partial improvement compared to their result for charge qubits. To make further progress, the detuning point for control should be more positive into the spin-like region with longer coherent times and thus Rabi oscillation is preferable. The approach for Rabi oscillations is to set the qubit in the spin-like region and apply MW bursts to rotate it around the *x* axis and vary the relative phase of successive MW bursts to rotate it around the *z* axis. An example of the Rabi oscillations is shown in Fig. 3h. In 2015, Kim *et al.* applied this method to the hybrid qubit and acquired a control fidelity of 93% for the *x* axis and 96% for the *z* axis [95]. This fully improved qubit quality compared to the charge qubit and simpler control method compared to the spin-1/2 qubit attracted a lot of attention to transplant the hybrid qubit into other systems. However, this qubit design relies on the valley–orbit states in silicon and thus cannot be borrowed directly. To address this problem, in 2016 Cao *et al.* implemented this qubit in a region with more electrons, (2, 3)–(1, 4) instead of (2, 1) and (1, 2), in a GaAs DQD [96]. The increased number of electrons allows the mixture of charge and spin degrees to be tuned freely such that the energy levels can be encoded like in a Si/SiGe DQD. Later, Wang *et al.* extended the hybrid qubit into a TQD. With an extra quantum dot for energy-level tuning, they realized a tunable operation frequency from 2 to 15 GHz, allowing a large range for frequency multiplexing [97]. In fact, the valley splitting in Si/SiGe quantum dots is not so controllable and varies from sample to sample. These new types of hybrid qubits are free of valley states and thus are more reproducible and scalable.

## TWO-QUBIT GATE IN SEMICONDUCTOR

In contrast to single-qubit gates, which all require two-axis control, the two-qubit gate can be realized in many different ways. In fact, the CNOT gate is not the only two-qubit gate for universal quantum computing. Others include the square root of the SWAP gate $( {\sqrt {{\rm{SWAP}}} } )$ and the controlled phase gate (CZ) [16]. The SWAP gate swaps the two-qubit state and the $\sqrt {{\rm{SWAP}}} $ gate performs half the way of such SWAP. The CZ gate acts on two qubits in such a way that a π rotation around the *z* axis is performed on the target qubit only when the control qubit state is $| 1 \rangle $. In the semiconductor quantum devices, these different two-qubit gates can also be divided into three different categories considering the source of interaction: exchange interaction, Coulomb interaction and circuit quantum electrodynamics (cQED). In the following subsections, we will introduce the realization of two-qubit gates using different types of interactions and discuss the progress.

### Exchange interaction

**Figure 4.**
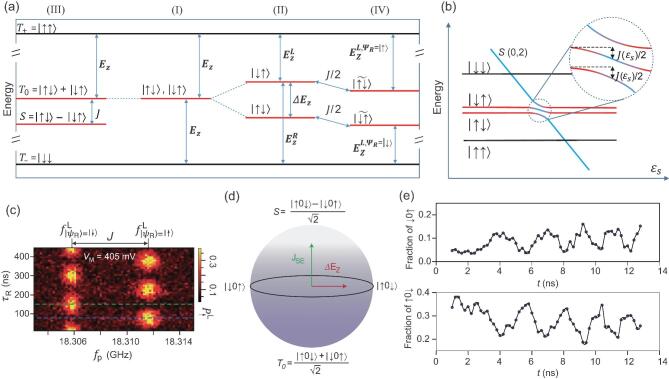
Two-qubit gates based on exchange interaction. (a) Eigenenergies of two spins in a DQD in the presence of a magnetic field gradient $\Delta {\boldsymbol E}_{\boldsymbol z}$ and relevant transitions between them for four distinct realistic parameter regimes: (I) both $\Delta {\boldsymbol E}_{\boldsymbol z}$ and $J$ equal zero; (II) only $\Delta {\boldsymbol E}_{\boldsymbol z}$ is non-zero; (III) both $\Delta {\boldsymbol E}_{\boldsymbol z}$ and $J$ are non-zero and $J$ is much bigger than $\Delta {\boldsymbol E}_{\boldsymbol z}$; and (IV) both $\Delta {\boldsymbol E}_{\boldsymbol z}$ and $J$ are non-zero and $J$ is much smaller than $\Delta {\boldsymbol E}_{\boldsymbol z}$. (b) Energy levels of two spin states as a function of detuning ${\varepsilon _s}$ in condition (IV). The energy shift $J({\varepsilon _s})/2$ of the antiparallel-spin states is denoted in the enlarged dotted circle. (c) The probability of spin-up states for the left qubit $P_ \uparrow ^{\rm{L}}$ as a function of the MW burst time τ_R_ and MW frequency${f_{\rm{p}}}$. The MW bursts are applied on the right qubit. Two resonance frequencies of the left qubit are split by $J$. (Adapted from [17].) (d) Bloch-sphere representation of the singlet–triplet subspace in the superexchange regime with control axes ${J_{{\rm{SE}}}}$ and $\Delta {\boldsymbol E}_{\boldsymbol z}$. (Adapted from [107].) (e) Observation of superexchange-driven spin oscillations. (Adapted from [107].)

Exchange interaction is a quantum mechanical effect for identical particles. In this context, it refers to the interaction between two spins. Two-qubit gates using exchange interaction have been proposed for spin-1/2 qubits [20], singlet–triplet qubits [62], exchange-only qubits [76] and hybrid qubits [91]. Among these, the exchange interaction between spin-1/2 qubits has been investigated most thoroughly in experiments and thus we mainly discuss it in the following. The interaction strength *J* and Zeeman energy difference $\Delta {{\boldsymbol{E}}_{\boldsymbol{z}}}$ are two competing factors in controlling two interacting spins, and their relative magnitude determines the energy levels of the system. Figure 4a depicts the energy-level spectrum in four different cases [98]: (I) When both $\Delta {{\boldsymbol{E}}_{\boldsymbol{z}}}$ and *J* equal zero, the qubit eigenstates are directly product states and all single spin-flip transitions are energetically degenerate. (II) If only $\Delta {\boldsymbol E}_{\boldsymbol z}$ is non-zero, two spins can be addressed at different transition frequencies and single spin qubit control can be achieved. (III) If $\Delta {\boldsymbol E}_{\boldsymbol z}$ and *J* are non-zero and *J* is much bigger than $\Delta {\boldsymbol E}_{\boldsymbol z}$, the two-qubit eigenstates are no longer effectively product states but singlet and triplets. This is just like the case of singlet–triplet qubits, and a $\sqrt {{\rm{SWAP}}} $ gate can be implemented with a π/2 rotation around the *z* axis. (IV) If $\Delta {\boldsymbol E}_{\boldsymbol z}$ and $J({\varepsilon _s})$ are non-zero and *J* is much smaller than $\Delta {\boldsymbol E}_{\boldsymbol z}$, the qubit eigenstates can still be viewed as product states with small corrections due to spin–charge hybridization. In this regime, each qubit transition frequency is no longer independent of the state of the other and thus permits CZ or CNOT operations.

For the $\sqrt {{\rm{SWAP}}} $ gate, it was first demonstrated by Petta *et al.* using GaAs quantum dots in 2005, reporting an operation on input state $| { \uparrow \downarrow } \rangle $ or $| { \downarrow \uparrow } \rangle $ with a time of 180 ps [44]. However, limited by the measurement method, they could not perform the $\sqrt {{\rm{SWAP}}} $ gate for other input states like $| { \uparrow \uparrow } \rangle $ or $| { \downarrow \downarrow } \rangle $. In 2011, Nowack *et al.* first demonstrated independent single-shot readout of two electron spins using energy-selective readout, and upon this result they measured the full truth table for a SWAP gate with four different input states [99]. In the same year, Brunner *et al.* combined the SWAP*^n^* gate (*n* means multiples of the operation time of a SWAP gate) with single-qubit rotations and demonstrated two-qubit entanglement [100].

For the CZ gate in semiconductor, it was first theoretically discussed by Meunier *et al.* in 2011 [101]. The energy levels as functions of detuning ${\varepsilon _s}$ are shown in Fig. 4b; a vanishing detuning lowers the antiparallel-spin states with $J({\varepsilon _s})/2$ and thus allows a phase shift of $J( {{\varepsilon _s}} ){t_{{\rm{wait}}}}/2$ when applying a detuning pulse for a fixed time ${t_{{\rm{wait}}}}$, resulting in a unitary transformation in the basis of $| { \uparrow \uparrow } \rangle $, $| { \uparrow \downarrow } \rangle $, $| { \downarrow \uparrow } \rangle $ and $| { \downarrow \downarrow } \rangle $:

(10) \begin{equation*} {U_{c{\rm{phase}}}} = \left( {\begin{array}{@{}*{4}{c}@{}} 1&0&0&0\\ 0&{{e^{iJ({\varepsilon _s}){t_{{\rm{wait}}}}/2}}}&0&0\\ 0&0&{{e^{iJ({\varepsilon _s}){t_{{\rm{wait}}}}/2}}}&0\\ 0&0&0&1 \end{array}} \right), \end{equation*}

when ${t_{{\rm{wait}}}}$ equals $\pi /J({\varepsilon _s})$, this gate control corresponds to a CZ gate only with additional single-qubit *z* rotations. The CZ gate was first demonstrated in a silicon MOS DQD by Veldhorst *et al.* in 2015 [102]. By combining it with two $\pi /2$ rotations, they implemented a CNOT gate and observed the corresponding anti-correlations. In 2018, Watson *et al.* used dynamical decoupling pulses to improve the performance of CZ gates and performed the Deutsch–Josza algorithm and the Grover search algorithm with a natural Si/SiGe DQD, suggesting the first implementation of a programmable two-qubit quantum processor [103]. The Bell-state tomography, which is a characterization of the two-qubit gate performance, indicated prepared state fidelities of 85–89%. Considering the state preparation and measurement (SPAM) errors brought about by the Bell-state tomography, they then used character randomized benchmarking to study the CZ gate control fidelity and obtained a value of ∼91% [104].

For the CNOT gate, it can be realized by directly driving the qubits via MW bursts for a time when *J* is non-zero [17,34]. As Fig. 4a suggests, MW bursts with a frequency resonant with the transition of $| { \downarrow \uparrow } \rangle $ to $| { \uparrow \uparrow } \rangle $ and off-resonant with other transitions can cause the left qubit to rotate only when the right qubit state is ${|}\!\! \uparrow \rangle $. As a result, the rotation of the left qubit is controlled by the right qubit state, and it corresponds to a CNOT gate when the controlled rotation angle equals π, as illustrated in Fig. 1b. Actually, the CNOT gate here has to be calibrated to eliminate the conditional phase caused by exchange interaction, and usually we call it a conditional rotation (CROT) gate. In experiments, *J* can be controlled by manipulating the detuning ${\varepsilon _s}$ or the inter-dot tunneling *t*. In Watson *et al.*'s experiment, they controlled ${\varepsilon _s}$ to implement a CROT gate for measuring a qubit state via another qubit [103]. In 2018, Zajac *et al.* realized a direct CNOT gate by controlling inter-dot tunneling *t*, reporting a Bell-state fidelity of 78% [17]. The device is shown in Fig. 1c, and they used the middle gate M to directly control the inter-dot tunneling and thereby the exchange interaction. When the interaction is turned on, the resonance frequency of the left qubit is dependent on the right qubit state. As illustrated in Fig. 4c, the response of the left qubit to MW bursts oscillates between two frequencies as the right qubit is under Rabi oscillation, and the two state-dependent resonance frequencies are separated by *J*. On top of that, the CROT gate can also be implemented with a constant *J*. With this new approach, in 2018, Huang *et al.* set up a new record with fidelity up to 98% via two-qubit randomized benchmarking based on a purified silicon MOS DQD [34].

In addition, except two nearest qubits, a two-qubit gate on the strength of exchange interaction can also be applied to a qubit with the next-nearest neighbor. This was investigated by a number of groups. In 2013, both Braakman *et al.* and Busl *et al.* found a direct tunnel coupling of two outer quantum dots of a TQD, suggesting that a superexchange interaction may exist between the two electron spins in the outer quantum dots [105,106]. With the empty central quantum dot acting as a mediator, in 2016, Baart *et al.* first demonstrated superexchange-interaction-driven oscillations of two distant spins in the outer quantum dots [107]. The Bloch-sphere representation of the *S–T_0_*states of the two outer spins and corresponding *S–T_0_*oscillations are shown in Fig. 4d and e, respectively. Similar to singlet–triplet qubits, the *z* axis of the Bloch sphere is controlled by the superexchange interaction ${J_{SE}}$ while the *x* axis is controlled by the Zeeman energy difference $\Delta {\boldsymbol E}_{\boldsymbol z}$. Moreover, a multi-electron quantum dot can also serve as a mediator for long-range exchange interaction and in 2018 Malinowski *et al.* demonstrated exchange oscillations of two distant spins using this method [108]. Another approach to realize coupling with a next-nearest neighbor is to shuttle a local entangled state to a farther quantum dot. In 2018, Nakajima *et al.* found that this approach can be assisted by dephasing noise and observed the corresponding coherent evolutions [109]. Apart from these, the exchange interaction can be applied to different types of qubits. In 2018, a CZ gate was implemented for a spin-1/2 qubit and a singlet–triplet qubit via a tunable inter-qubit exchange coupling by Noiri *et al.* [110] in a GaAs TQD.

As for donor spin-1/2 qubits, the exchange coupling is not that easy to harness because of its rigid requirement of the atomic-scale precision of the two donors. Some progress was recently made in 2018 with the observation of anti-correlated spin states between two donors in silicon separated by 16 ±1 nm [111]. The small tunnel coupling in their device prohibited measurement of coherent oscillations, indicating that a much smaller separation is needed for further research. To resolve this challenge, a series of two-qubit gate strategies with slightly relaxed requirements on donor placement were proposed, including taking advantage of hyperfine interaction [112] and magnetic dipole–dipole coupling [35], or introducing an intermediate coupler such as probe spin [36] and quantum dots [37]. Moreover, the recent proposed flip-flop qubit promises a two-qubit gate that can be implemented at separations of hundreds of nanometers with the second-order electric dipole–dipole interaction [113]. Nonetheless, there is still a long way ahead before these proposals become reality.

### Coulomb interaction

Coulomb interaction is the electrostatic coupling between two or more electrons. The two-qubit gate based on Coulomb interaction has been proposed for singlet–triplet qubits [114], exchange-only qubits [115], hybrid qubits [92] and charge qubits [116]. So far, both experiments on singlet–triplet qubits and charge qubits have been demonstrated.

**Figure 5.**
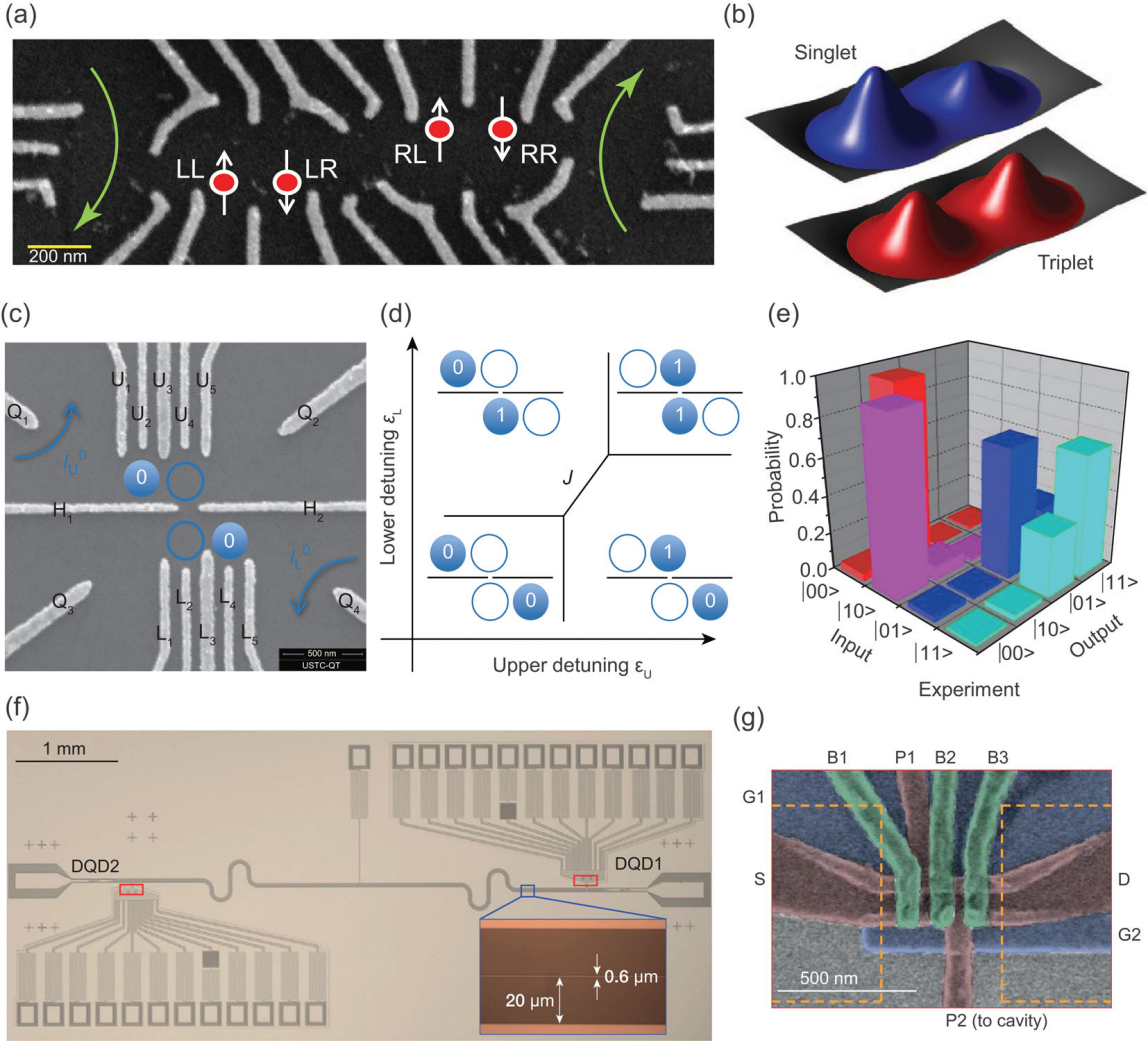
Two-qubit gates based on Coulomb interaction and circuit quantum electrodynamics (cQED) with a DQD. (a) SEM image of a device for entangling two singlet–triplet qubits. The approximate locations of the electrons in the two qubits are denoted by red circles with arrows. The current paths for the SETs are denoted by green arrows. (Adapted from [118].) (b) Schematic of the charge configuration for singlet (blue) and triplet (red). (Adapted from [118].) (c) SEM image of a device for coupling two charge qubits. The solid blue circles denote the charge configuration for the corresponding state and two current paths for QPCs are denoted by blue arrows. (Adapted from [120].) (d) Diagram showing Coulomb-interaction-induced $J$ as a function of detuning for the upper DQD ${\varepsilon _U}$ and lower DQD ${\varepsilon _L}$. (Adapted from [120].) (e) Probabilities for the output states of a CROT operation acquired experimentally by preparing qubits in different input states ${|00} \rangle ,| {10} \rangle ,| {01} \rangle $ and ${|11} \rangle $. (Adapted from [120].) (f) Optical image of the superconducting resonator coupling two DQDs. The inset shows an optical image of the center pin and vacuum gap. (Adapted from [127].) (g) False-color SEM image of a DQD with gate P2 coupled to the resonator. The micro-magnets for spin–photon coupling are indicated by orange dashed lines. (Adapted from [127].)

For singlet–triplet qubits, the two-qubit gate was first experimentally investigated by Weperen *et al.* in 2011 using two electrostatically coupled DQDs [117]. They found that the precession frequency of the singlet–triplet qubit can be controlled by the charge arrangement of an electrostatically coupled DQD. Then, in 2012, Shulman *et al.* utilized the different charge occupations of *S* and *T*_0_ to control the precession frequency of another qubit [118]. A typical device and a schematic of the charge configurations of *S* and *T*_0_ are shown in Fig. 5a and b, respectively. The Hamiltonian can thus be given by

(11) \begin{eqnarray*} {{\boldsymbol{H}}_{2ST}} &=& ({J_1}\left( {{\sigma _z} \otimes I} \right) + {J_2}\left( {I \otimes {\sigma _z}} \right))/2 \nonumber\\ &&\!\! +\, (\Delta {{\boldsymbol{E}}_{{\boldsymbol{z}},1}}( {{\sigma _z} \!\otimes \!I} ) \!+\! \Delta {{\boldsymbol{E}}_{{\boldsymbol{z}},2}}( {I \!\otimes \!{\sigma _z}} ))/2 \nonumber\\ &&\!\!+ {J_{12}}( {{\sigma _z} - I} ) \otimes ( {{\sigma _z} - I} )/4. \end{eqnarray*}

Here *I* is the identity operator, *J_i_* and $\Delta {{\boldsymbol{E}}_{z,i}}$ are the exchange splitting and Zeeman energy difference, with *i* =1,2 referring to the corresponding qubit, and *J*_12_ is the two-qubit coupling strength. When rotating both qubits around the *z* axis simultaneously, the state-dependent charge occupation will mediate a coupling strength ${J_{12}} \propto {J_1}{J_2}$, and an entangled state may be produced with a certain operation time. With dynamical decoupling pulses, they implemented an entangling gate in this way and measured the generated state, yielding a Bell-state fidelity of 72%. Here, the entangling gate is equivalent to a CZ gate up to single-qubit rotations. Using a dominating magnetic gradient to suppress charge noise, Nichol *et al.* reported an entangling gate fidelity of 90% in 2017 [71].

For charge qubits, the two-qubit gate was first investigated by Petersson *et al.* [119] and Shinkai *et al.* [116] in 2009. The level structure of the interacting two-qubit system was probed and correlated oscillations were observed. In 2015, on the basis of these results, Li *et al.* demonstrated a CROT gate of two strongly coupled charge qubits [120]. The two-qubit Hamiltonian can be written as

(12) \begin{eqnarray*} {H_{2C}} &=& ({\varepsilon _1}\left( {{\sigma _z} \otimes I} \right) + {\varepsilon _2}\left( {I \otimes {\sigma _z}} \right))/2 \nonumber\\ &&+ {t_1} \left( {{\sigma _x} \otimes I} \right) + {t_2}\left( {I \otimes {\sigma _x}} \right) \nonumber\\ &&+ {J_{12}}\left( {{\sigma _z} - I} \right) \otimes \left( {{\sigma _z} - I} \right)/4. \end{eqnarray*}

Here ${\varepsilon _i}$ and ${t_i}$ are the detuning and inter-dot tunneling, with $i = 1,2$ referring to the qubit, and *J*_12_ is again the two-qubit coupling strength. A typical device [120] is depicted in Fig. 5c, showing two electrostatically coupled GaAs DQDs. Charge qubits are formed in each DQD and can be controlled independently by detuning. Figure 5d shows the interaction between two charge qubits when controlling the detuning. The zero detuning point for the upper qubit, i.e. the anti-crossing point for the qubit to change from $| 0 \rangle $ to $| 1 \rangle $, will shift to a higher point by an amount *J*_12_ when the lower qubit state is changed from $| 0 \rangle $ to $| 1 \rangle $. Here we denote the lower point as $\varepsilon _U^{{\Psi _L} = \ | 0 \rangle \ }$ and the higher point $\varepsilon _U^{{\Psi _L} = \ | 1 \rangle \ }$. A CNOT gate can thus be applied by pulsing the upper qubit to $\varepsilon _U^{{\Psi _L} = \ | 0 \rangle \ }$ so that the upper qubit will be rotated only if the lower qubit state is $| 0 \rangle $. In this way, they measured the truth table (see Fig. 5e) of a CROT gate and extracted a control fidelity of ∼68%. In 2016, Ward *et al.* also demonstrated controlled rotations for two charge qubits in two coupled Si/SiGe DQDs [121]. To take a step further, in 2018, Li *et al.* demonstrated three-qubit controlled rotations using three coupled GaAs DQDs [122], which is a first attempt to go beyond the two-qubit limit in semiconductor devices and suggests that semiconductor qubits are amenable to large-scale manufacture.

### Coupling to the resonator

In addition to proximity coupling like exchange interaction and Coulomb interaction, semiconductor qubits can also be coupled distantly through cQED [123,124]. In 2012, both Frey *et al.* and Petersson *et al.* demonstrated a DQD dipole coupled to an on-chip distributed superconducting resonator [125,126]. An experiment set-up and the corresponding DQD with a surface electrode connected to the resonator are shown in Fig. 5f and g, respectively [127]. For the cQED, a Hamiltonian of the Jaynes–Cummings type can be given as

(13) \begin{eqnarray*} {{\boldsymbol{H}}_{DC}} &=& {\omega _c}(n + 1/2) + {\omega _q}{\sigma _z}/2 \nonumber\\ &&+ {g_c}\sin \theta({a^\dagger }{\sigma ^ - } + a{\sigma ^ + }). \end{eqnarray*}

Here the first term refers to the photon of the resonator with photon frequency ${\omega _c}$ and photon number operator *n*, the second term refers to the DQD with transition frequency ${\omega _q} = \sqrt {\varepsilon _c^2 + 4{t^2}} ,$ the third term refers to the photon–DQD coupling with coupling rate ${g_c}$, the creation and annihilation operators of photon ${a^\dagger }$ and *a*, the Pauli operators ${\sigma ^ - }$ and ${\sigma ^ + }$, and $\sin \theta = 2t/\sqrt {\varepsilon _c^2 + 4{t^2}} $. A change of detuning ${\varepsilon _c}$ and tunneling *t* of the DQD will alter the Hamiltonian and cause a modification of photons in the resonator, resulting in a phase and amplitude change in the transmitted or reflected signal, which can be observed in experiments. It is noteworthy that the SQD can also be coupled to a resonator [128]; due to space limitations we will not go into detail about its mechanism.

Using photons in the resonator as a mediator, distant coupling of two DQDs is thus expected. Since 2012, several groups have reported non-local coupling between distant quantum dots using a resonator, including Delbecq *et al.* [128] and Deng *et al* [129]. However, the relatively strong atomic (qubit) dephasing rate $\Upsilon $, which is inversely proportional to the dephasing time, and photon loss rate κ impeded their step for two-qubit gate control. To overcome this challenge, a strong coupling regime in which ${g_c}$ exceeds κ and $\Upsilon $is in demand. Since 2016, the strong coupling of a resonator to a DQD in the Si/SiGe heterostructure [130], the GaAs/AlGaAs heterostructure [131] and the carbon nanotube [132] have been reported successively. Upon these results, in 2018, Nicolí *et al.* [133] demonstrated tunable photon-mediated coupling of two charge qubits and measured the two-qubit coupling strength. In the meantime, Scarlino *et al.* [134] also demonstrated coherent coupling between a semiconductor charge qubit and a superconductor qubit, and even observed controlled oscillations. On top of these results, strong spin–photon coupling was realized in 2018 by several groups [127,135,136]. Further exploration of coherent coupling and even a two-qubit gate of spin-1/2 qubits [137], singlet–triplet qubits [138] and resonant exchange qubits [139] is thus expected.

## CHALLENGES AND OPPORTUNITIES

The developments and recent advances of high-fidelity single- and two-qubit control in semiconductors have been introduced above, indicating a strand of a scalable fault-tolerant quantum computing fabric. However, there are still some challenges to resolve before the next leap. Here, we discuss these challenges and introduce some research progress.

### Readout of qubits

As discussed above, the readout method of most types of semiconductor qubits depends on a proximate charge sensor. Once the charge state of the quantum dot or a donor changes, the resistance of the charge sensor will change accordingly. Therefore, the readout speed and fidelity are directly related to the bandwidth and signal-to-noise-ratio (SNR) of the charge sensor. To include both the bandwidth and SNR, in the following we use the charge sensitivity to characterize the performance of a readout method:

(14) \begin{eqnarray*} &&{\rm{Charge\ sensitivity\ }}\nonumber\\ &&= 1/(\left( {{\rm{SNR}}} \right) \cdot \sqrt {{\rm{Bandwidth}}} )(e/\sqrt {{\rm{Hz}}} ).\nonumber\\ \end{eqnarray*}

A typical state-of-the-art charge sensor with a transconductance amplifiuer at room temperature (RT) can achieve a charge sensitivity down to 820 $\mu e/\sqrt {{\rm{Hz}}} $ for a 30 kHz bandwidth [140]. To improve its performance, several approaches have been investigated [141].

**Figure 6.**
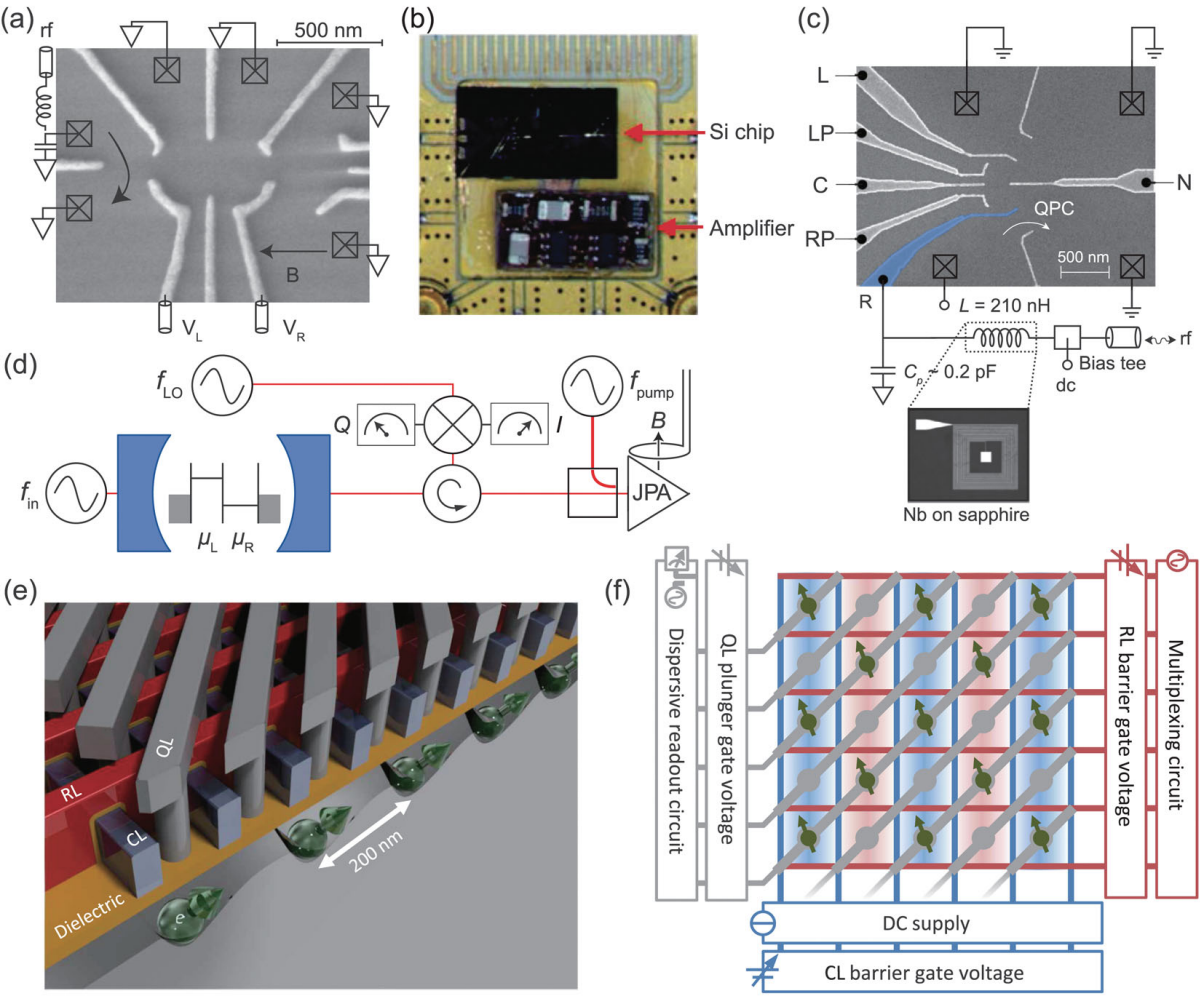
Approaches to improve readout quality and a crossbar network for large-scale integration. (a) SEM image of a device using an rf-QPC, indicating ohmic contacts (boxes), fast gate lines, impedance-matching circuit, grounded contacts, and the external magnetic field direction. (Adapted from [145].) (b) Picture of a silicon device with an adjacent cryogenic amplifier circuit mounted on printed circuit board. (Adapted from [150].) (c) False-color SEM image of a device using an rf gate sensor. One electrode (blue) is coupled directly via a bondwire to an off-chip Nb/Al_2_O_3_ superconducting lumped-element resonator. (Adapted from [152].) (d) Diagram of the device using a distributed superconducting resonator with the input field ${f_{{\rm{in}}}}$. The output field is sent to a JPA through a circulator and then demodulated into the *I* and *Q* quadratures with a local oscillator tone ${f_{{\rm{LO}}}}$. A directional coupler is used to couple the pump field at frequency $\ {f_{{\rm{pump}}}}$ (Adapted from [158].) (e) and (f) are a 3D model and schematic representation of the crossbar network for a 2D quantum-dot array. CLs (blue), RLs (red), and QLs (gray) connect the qubit grid to outside electronics for control and readout [39].

The first approach is to couple an impedance-matching radio frequency (rf) resonant circuit to the integrated charge sensor, usually a SET or a QPC, to form an rf-SET [142] or an rf-QPC [143,144]. Its operating principle is to detect the modulation of the reflected or transmitted rf signal by resistance change of the charge sensor. The impedance-matching network lowers the high resistance of the charge sensor, usually >50 kΩ, towards the characteristic impedance of a transmission line ∼50 Ω. Thus the RC time constant of the circuit is reduced and thereby the working bandwidth is improved. The first demonstration of using an rf-SET to detect charge states in semiconductor was in 2003, when Lu *et al.* fabricated an aluminum rf-SET to detect real-time electron tunneling in a GaAs quantum dot [142]. In 2007, Reilly *et al.* and Cassidy *et al.* reported the characterization of rf-QPCs fabricated using the GaAs/AlGaAs heterostructure [143,144]. Both the rf-SET and the rf-QPC can offer a charge sensitivity lower than 200 $\mu e/\sqrt {{\rm{Hz}}} $ with a bandwidth over 1 MHz. For the applications in qubit readout, in 2009, Barthel *et al.* used an rf-QPC to detect a singlet–triplet qubit and reported a single-shot measurement with fidelity over 90% for a bandwidth ∼143 kHz [145]. The rf-QPC that they used is depicted in Fig. 6a, with an ohmic contact (box) coupled to an impedance-matching network formed by an inductor and a parasitic capacitance of the bond pads and wires. In 2010, by using a GaAs quantum-dot-based rf-SET, they even improved the measurement bandwidth to 10 MHz for the readout of a singlet–triplet qubit [146]. Beyond that, this technique also applies to other types of qubits such as charge qubits [147], spin-1/2 qubits [18] and exchange-only qubits [77].

The second approach is to use cryo-amplifiers. For conventional measurement methods, the readout bandwidth is also limited by the transconductance amplifier at room temperature (RT). When the readout bandwidth is increased, the RT amplifier gain will decrease and so does the SNR. In fact, the SNR can still be increased if the amplification is introduced at a lower temperature before the dominant noise comes in. Inspired by this idea, several attempts have been made to fabricate a suitable cryo-amplifier located much closer to the device, including employing a high-electron-mobility transistor (HEMT) [148] and a SiGe heterojunction bipolar transistor (HBT) [149]. In 2016, Tracy *et al.* demonstrated the single-shot readout of a P-donor-electron spin-1/2 qubit with 96% visibility of Rabi oscillations by using a cryogenic two-stage HEMT circuit adjacent to the qubit device [150], as shown in Fig. 6b. For a bandwidth of 100 kHz, they achieved an SNR of 9 and a charge sensitivity of 300 $\mu e/\sqrt {{\rm{Hz}}} $, nearly twice as good as the values of the state-of-the-art charge sensor with an RT amplifier.

Unlike the approaches mentioned above, the third approach gets rid of a charge sensor by coupling the surface electrode of a DQD directly to a resonator. This mechanism was introduced in the section entitled ‘Two-qubit gate in semiconductor'. For experiments, in 2010, Petersson *et al.* first demonstrated a lumped-element resonator circuit coupled to the reservoir of a DQD in GaAs and used it to probe charge and spin states [151]. Then, in 2013, Colless *et al.* coupled the lumped-element resonator to a gate electrode of a DQD and named it an rf gate sensor [152]. An rf gate sensor is shown in Fig. 6c, with a lumped-element resonator constituted by an inductor *L* ∼ 210 nH and a parasitic capacitance *C_p_* ∼ 0.2 pF. In 2015, Gonzalez-Zalba *et al.* demonstrated a record sensitivity of 37 $\mu e/\sqrt {{\rm{Hz}}} $ for a bandwidth ∼ 1 kHz with a gate sensor [153] for silicon SOI quantum dots. This value was further improved to 1.3$\mu e/\sqrt {{\rm{Hz}}} $ in 2018 [154] for a bandwidth of ∼10 Hz. Single-shot readout of singlet–triplet qubits using rf-gate sensors was realized by Pakkiam *et al.*, Urdampilleta *et al.* and West *et al.* in 2018 with donors in silicon, a silicon MOS DQD and a silicon SOI DQD [155–157], respectively. Among them, the best reported readout fidelity is 99.7% for a 1 kHz bandwidth.

Alternatively, the lumped-element resonator can be replaced by an on-chip distributed superconducting resonator, like the experiments for two-qubit gates. Early in 2012, Petersson *et al.* first demonstrated readout of a spin-1/2 qubit using a superconducting resonator based on spin blockade [126]. Also, in 2015, Stehlik *et al.* added a Josephson parametric amplifier (JPA) at the output of the resonator to amplify the signal, as shown in Fig. 6d, resulting in a charge sensitivity of 80 $\mu e/\sqrt {{\rm{Hz}}} $ for a bandwidth of 2.6 MHz [158]. Later, Mi *et al.* replaced the JPA for a traveling-wave parametric amplifier (TWPA) and demonstrated strong coupling of the DQD to a resonator [130]. With the help of a slanting magnetic field generated by a micro-magnet, in 2018, they further demonstrated strong spin–photon coupling and performed dispersive readout of a spin-1/2 qubit [127]. For charge qubits, in 2017, Scarlino *et al.* [159] demonstrated dispersive readout of a charge qubit and measured an intrinsic dephasing time *T*_2_ up to (43.1±4.3) ns. Furthermore, coupling to resonators not only requires no charge sensor, but also allows frequency multiplexing. Since 2014, the proposals of multiplexing readout of spin and charge qubits have been demonstrated for larger-scale applications [160,161].

### Material developments

The semiconductor qubit has long been argued for excellent scalability considering the success of semiconductor technology for classical computers. However, the largest number of qubits controlled in the same device so far is still no more than four [162]. This can be partially explained by the limited experimental conditions of colleges in contrast to the industry, but more importantly, the properties of the substrates play a key role in fabrication. Traditional modulation-doped GaAs/AlGaAs heterostructure [21] is an excellent substrate for quantum dots owing to its relatively high mobility and steady quality. However, the nuclear noise in GaAs hinders the research on high-fidelity spin qubits. To eliminate spin noise, researchers have to move to another substrate: silicon [17,103] or even purified silicon with less Si^29^ [18,53,102]. Nevertheless, there are many challenges to the implementation of qubits in silicon, such as complicated valley degeneracy and elaborate control of the small electronic wave function [22].

The valley degeneracy originates from the six degenerate minima of the conduction band of bulk silicon and these sub-bands are termed valleys. For donors in silicon, the additional degeneracy of the valley state is not a concern since the strong confinement potential from the dopant atom can lift it easily. However, for silicon quantum dots, although the four in-plane valleys are raised far away from the two out-of-plane valleys, the small splitting of the two low-lying valleys still complicates the qubit control [22]. For spin-1/2 qubits, it has been observed that spin–valley mixing can cause a sizable decrease of spin lifetime [163], the *g*-factor is renormalized by SOC with valley states [164], the probability of occupying the valley state will deteriorate spin initialization, and inter-valley scattering may limit the dephasing time [52]. For singlet–triplet qubits and exchange-only qubits, the lower valley may lift spin blockade and reduce the readout fidelity [68], and for hybrid qubits in silicon quantum dots, the energy scale caused by the valley splitting should be controlled in a reasonable range. Therefore, reproducible and controllable valley splitting in silicon is in demand. So far, several studies have been performed on valley splitting in silicon [163,165–169]. As a whole, the valley splitting in quantum dots based on silicon MOS and SOI is in the range of 300–800 μeV and 610–880 μeV, respectively, and can be easily controlled by electric field [163,168]. Recent research even suggested that the single-electron valley splitting in silicon MOS quantum dots is both tunable and predictable; thus silicon MOS is a promising platform for qubit control [166]. In contrast, the valley splitting in Si/SiGe quantum dots has been found to vary considerably from sample to sample with a range of only 35–270 μeV [167]. The origins may be attributed to the interfacial steps and atomic-scale disorder of the Si well; such disorder mainly arises from the substrate and relaxed buffer [169]. To further control the valley splitting in a reproducible way, other research on how to control such disorder is needed.

For the small electronic wave function, a much smaller lithographical size of the quantum dot is required for fine control. The traditional depletion-mode gate design used in GaAs quantum dots, as shown in Fig. 3a, d and g, is like a big conference hall and the formed quantum dots are like conferees; this therefore puts an obstacle in the way of precise control. In contrast, a new overlapping gate design that accumulates electrons only under small electrodes with a diameter on the similar scale of a quantum dot is preferred. This new design was first introduced by Angus *et al.* in 2007, who used two layers of aluminum electrodes with local insulator in between to define a quantum dot in silicon MOS, resulting in a lithographical diameter as small as 50 nm [170]. It is worth noting that the lithographical size of the quantum dot refers to the surface area defined by the electrodes and is not equal to the actual size of the quantum dot formed by the electric field. The lithographical diameter in silicon MOS was later reduced to 30–40 nm for controlling single electrons [171,172]. For quantum dots in the Si/SiGe heterostructure, similar electrode designs were demonstrated by Zajac *et al.* [173] and Borselli *et al.* [174] in 2015. One device fabricated by Zajac *et al.* is shown in Fig. 1c with three layers of electrodes, which are confinement gates (blue), plunger gates (red) and barrier gates (green), to form a DQD in the upper channel and a SET in the lower channel. In contrast to silicon MOS devices, the electrode size to form a quantum dot in Si/SiGe heterostructure can be a little larger, usually ∼80 nm. This less-stringent requirement for lithography allows a great improvement in the reproducibility and scalability of quantum dots. In 2016, Zajac *et al.* presented a device containing a linear array of nine quantum dots with reproducible properties and three quantum dots as SETs [140]. Using a device with the same electrode structure, in 2018, Mills *et al.* demonstrated shuttling a single charge across this linear array [175]. In contrast, the largest number of working quantum dots in a silicon MOS device is still no more than four [53,172], implying the difficulty set by lithography. However, other designs including a single-layer electrode layout using poly-Si in silicon MOS [176] and silicon quantum dots fabricated using SOI technology have also been developed [55], implying alternatives for reproducibility and scalability.

Beyond silicon, germanium, which can host the aforementioned hole-spin qubits, is also a promising material for high-fidelity qubit control. Like silicon, it can be isotopically purified to improve coherence times. Also, it is free of valley degeneracy and thus supports the reproducibility of well defined qubits. Recent reports on quantum dots in Ge hut wires and the Ge/GeSi heterostructure have shown some initial results based on this material [56,60,177]. Further in-depth research is still needed to implement high-fidelity single- and two-qubit gates in such material and verify their homogeneity and reproducibility.

### Scalable design

Now that high-fidelity control and readout of single- and two-qubit gates in semiconductor have been demonstrated, the next challenge lies in how to scale them to tens and hundreds of qubits. The corresponding constraints and problems have been investigated thoroughly since 2015, including the geometry and operation time constraints [178,179], engineering configuration for the quantum–classical interface [180–183], and even the quantifying of system extensibility [184]. In the light of these discussions, several proposals for scaling up have been proposed, varying from the crossbar network [38,39] for spin-1/2 qubits in silicon MOS quantum dots, the 2D lattice of donor qubits in silicon [35,36], to hybrid architecture like donor–dot structure [37] and flip-flop qubit structure [113].

In these proposals, the key issue is the wiring strategy of readout lines and control lines for single- and two-qubit gates as well as the balance between feasibility and high-quality performance. Here, we take the crossbar network of silicon spin-1/2 qubits as an example to illustrate these considerations. In Li *et al.*'s work [39], as Fig. 6e and f shows, three successive layers that play the roles of column barrier lines (CL), row barrier lines (RL), and diagonal plunger lines (PL) form a 2D array. Successively tuning CLs, RLs and PLs, electrons can be loaded from reservoirs at the array boundary into the qubit array for single-electron occupation. Moreover, the CLs also carry direct currents to generate a magnetic field gradient $\Delta {\boldsymbol E}_{\boldsymbol z}$ for adjacent columns, while the QLs are connected to a dispersive readout circuit to play the role of gate sensors. To perform single-qubit rotations, global superconducting striplines above the chip are used to provide in-plane oscillating magnetic fields. A $\sqrt {{\rm{SWAP}}} $ gate can be performed by two spins in the adjacent rows of the same column without $\Delta {\boldsymbol E}_{\boldsymbol z}$, while the spin–charge conversion relies on the two spins in the adjacent columns of the same row with $\Delta {\boldsymbol E}_{\boldsymbol z}$, which can constitute a spin-blockade regime with QLs to probe the tunneling event. Qubits can be moved freely along the rows and columns of the grid to perform a two-qubit gate or readout with the help of spin shuttling. Also, the spin shuttling can be used to add a controllable phase for a single qubit, thus a rotation around the *z* axis is achieved without extra control. However, obviously, the local control of one location may cause unwanted side effects in another location owing to the shared control property of these lines [185]. Another previous design [38] that connects every quantum dot in the grid directly via floating gates and vertical transistors can alleviate this problem. However, as a trade-off, the mediating floating gates and vertical transistors require more extensive manufacturing developments. More than that, there is still a gap between the proposed architectures and the reproducible quantum dots in current experiments. For example, the proposed dot tuning and charge sensing in a 2D grid have not been demonstrated simultaneously in experiments. For future advances, more experiments are needed to fill the gap.

## CONCLUSION AND OUTLOOK

In summary, we have introduced single- and two-qubit control of different types of semiconductor qubits and discussed their developments and recent groundbreaking progress. It should be noted that the developments of semiconductor qubits are very fast and there are also other new types of semiconductor qubits that are in proposal or under development. We cannot include them all due to the limitations of the authors' knowledge and space, but we still hope that this review can provide a clear picture of the state-of-the-art work. For quantum computation, we also discuss the challenges and new opportunities in this field, including new readout methods, material developments and scalable designs. These are the issues most considered in research now and the corresponding research progress may determine the future directions to some extent. Since superconducting circuits and ion traps have been sought after by industry for implementing a quantum computer, semiconductor quantum computation is also getting more and more attention by companies and governments in many countries. In fact, several groups have shown their ambition to further optimize semiconductor qubit quality for large-scale integration and scale it up to ten or more qubits in the next ten years, such as the Vandersypen and Veldhorst groups at TU Delft in the Netherlands with the help of Intel, the Simmons group at the Centre for Quantum Computation and Communication Technology (CQC2T) in Australia, the French Alternative Energies and Atomic Energy Commission (CEA) in France, etc.

In 2018, Professor John Preskill pointed out that noisy intermediate-scale quantum (NISQ) technology will be available in the near future [186]. This terminology refers to a quantum computer with 50–100 qubits that can surpass the capabilities of today's classical computers [187] and also a low circuit depth that is limited by insufficient control fidelity. Although they will not change the world immediately, the short-term applications for NISQ devices will still be consequential and could bring new opportunities for research and business. From this point of view, in addition to further improving the qubit quality and striving for fault-tolerant quantum computing in the coming years, the research into new applications for semiconductor devices is also important. Therefore, we anticipate that semiconductor quantum devices may develop fast and have a great impact on our lives from now on.
